# SNHG5 enhances colorectal cancer metastasis through RNA–protein interaction with GNB2 and activation of canonical Wnt signaling

**DOI:** 10.1016/j.ncrna.2025.12.002

**Published:** 2026-01-02

**Authors:** Xinyi Chen, Mu Yang, Xiaoxiao Luo, Xianglin Yuan

**Affiliations:** Department of Oncology, Tongji Hospital, Tongji Medical College, Huazhong University of Science and Technology, Wuhan, Hubei, China

**Keywords:** Colorectal cancer, Liver metastasis, SNHG5, GNB2, Wnt/β-catenin signaling, RNA–protein interaction, Long non-coding RNA, Bioinformatics analysis, Bioinformatics databases

## Abstract

**Background and purpose:**

Colorectal cancer (CRC) is one of the most prevalent and lethal malignancies worldwide, with distant metastasis—particularly to the liver—representing the primary cause of poor prognosis. Long non-coding RNAs (lncRNAs) have emerged as critical regulators of CRC progression, yet the mechanisms by which they modulate G protein signaling during hepatic metastasis remain unclear. This study aimed to determine the role of the lncRNA SNHG5 in CRC liver metastasis and to explore whether G protein–related mechanisms are involved in this process.

**Methods:**

We established murine MC38 CRC sublines with distinct metastatic capacities (F0 and F3) and performed RNA sequencing to identify key lncRNAs. Biotin-labeled RNA pull-down coupled with mass spectrometry was used to identify SNHG5-interacting proteins. The SNHG5–GNB2 interaction was validated using RIP, RNA-FISH, and Western blot analyses. Functional rescue assays, in vivo liver metastasis models, and Wnt pathway activity measurements were conducted to delineate downstream effects. Public transcriptomic datasets from GEO and TCGA were used to assess the expression patterns and prognostic relevance of SNHG5 and GNB2 in CRC and metastatic lesions.

**Results:**

SNHG5 was significantly upregulated in the highly metastatic F3 subline and predominantly localized in the cytoplasm. Pull-down and proteomic analysis identified GNB2, a classical G protein β-subunit, as a direct binding partner of SNHG5. Functionally, SNHG5 promoted cell proliferation, migration, epithelial–mesenchymal transition (EMT), and suppressed apoptosis, while GNB2 overexpression partially rescued the tumor-suppressive phenotypes induced by SNHG5 silencing. Mechanistically, the SNHG5–GNB2 axis enhanced Wnt/β-catenin signaling via increased p-GSK3β and β-catenin levels, thereby driving EMT. Transcriptomic analyses further revealed that GNB2 is upregulated in CRC and liver metastases and is associated with poor prognosis. Multi-omics data suggested additional roles for this axis in immune evasion, metabolic reprogramming, and remodeling of the metastatic microenvironment.

**Conclusion:**

This study provides the first evidence that SNHG5 promotes CRC liver metastasis through direct interaction with GNB2 and subsequent activation of the Wnt/β-catenin pathway. The SNHG5–GNB2 axis orchestrates a multilayered regulatory network that integrates EMT induction, immune suppression, and metabolic adaptation, highlighting its potential as a mechanistic driver and therapeutic target in metastatic CRC.

## List of abbreviations

AKT: protein kinase B
CAF: cancer-associated fibroblast
ceRNA: competing endogenous RNA
CRC: colorectal cancer
CRLM: colorectal liver metastasis
DC: dendritic cell
EMT: epithelial–mesenchymal transition
Fn14: fibroblast growth factor-inducible 14
GDF15: growth differentiation factor 15
GNB2: G protein subunit beta 2
GPCR: G protein-coupled receptor
HCC: hepatocellular carcinoma
LC–MS/MS: liquid chromatography–tandem mass spectrometry
lncRNA: long non-coding RNA
MAPK: mitogen-activated protein kinase
PD-1: programmed cell death protein 1
PD-L1: programmed death-ligand 1
PI3K: phosphatidylinositol 3-kinase
RIP: RNA immunoprecipitation
RNA-FISH: RNA fluorescence in situ hybridization
ROS: reactive oxygen species
S1P: sphingosine-1-phosphate
SNHG5/Snhg5: small nucleolar RNA host gene 5
STAT3: signal transducer and activator of transcription 3
TGF-β: transforming growth factor beta
Th17: T helper 17
TME: tumor microenvironment
TNF: tumor necrosis factor
Treg: regulatory T cell
Wnt: wingless-related integration site
Wnt/β-catenin: canonical Wnt/β-catenin signaling pathway

## 1 Introduction

Colorectal cancer (CRC) ranks as the third most commonly diagnosed malignancy and remains one of the leading causes of cancer-related mortality worldwide [[1], [2], [3], [4]]. According to global cancer statistics from 2020, CRC accounted for approximately 1.9 million new cases and 935,000 deaths, representing nearly 10 % of all cancer-related fatalities [1,2,5]. The predominant cause of death in CRC patients is distant metastasis, with the liver being the most frequent site; approximately 50 % of CRC patients develop liver metastases during the disease course [2]. Prognosis for patients with colorectal liver metastases (CRLM) is poor. Only 10–20 % of patients are eligible for surgical resection, with a 5-year survival rate of ∼30 % following surgery. For those with unresectable disease, 5-year survival drops to below 15 % [1,6]. Despite advances in systemic chemotherapy and surgical approaches, the overall prognosis of advanced CRC remains unsatisfactory [2]. This underscores the urgent need to elucidate the molecular mechanisms underlying CRC metastasis and to identify novel therapeutic targets to improve outcomes in patients with metastatic disease [7].

Tumor metastasis is a complex, multistep biological process. It typically begins with epithelial–mesenchymal transition (EMT), through which primary tumor cells acquire migratory and invasive properties. EMT-activated cells subsequently disrupt the basement membrane, invade the surrounding stroma, intravasate into blood or lymphatic vessels, survive in the circulation, extravasate, and ultimately colonize distant organs [[8], [9], [10]]. In CRC, EMT-mediated phenotypic plasticity and enhanced invasiveness are regarded as pivotal drivers of liver metastasis formation [11]. Recent studies have shown that cytokines in the tumor microenvironment (TME) can directly induce EMT. For example, Th17 cells secrete tumor necrosis factor–like weak inducer of apoptosis (TWEAK), which binds Fn14 receptors on tumor cells to directly promote EMT and accelerate CRC liver metastasis [12]. Similarly, growth differentiation factor 15 (GDF15) induces EMT-related gene expression in CRC cells and facilitates tumor invasion and dissemination [13]. Beyond EMT induction, the TME plays a central role in preparing the metastatic niche. Bone marrow–derived myeloid cells, macrophages, and fibroblasts release cytokines and exosomes that reshape the premetastatic microenvironment and support the colonization of circulating tumor cells at distant sites [14]. The canonical Wnt/β-catenin pathway exerts multiple oncogenic functions during CRC liver metastasis. On one hand, it promotes EMT by upregulating key transcription factors (Snail, ZEB1) and repressing E-cadherin [15]. On the other hand, it remodels the immune landscape to facilitate immune evasion by impairing dendritic cell maturation, promoting regulatory T cell (Treg) accumulation, excluding CD8^+^ T cell infiltration, and upregulating tumor PD-L1 expression—thus blunting antitumor immunity [16,17]. Furthermore, Wnt/β-catenin activation reprograms cellular metabolism through c-Myc–driven upregulation of glycolytic enzymes (PKM2, LDHA) and glucose transporters, thereby enhancing aerobic glycolysis (Warburg effect) and supporting metabolic adaptation during liver colonization [18]. Collectively, EMT, immune suppression, and metabolic reprogramming converge under canonical Wnt/β-catenin signaling to promote aggressive CRC metastasis. Elucidating the upstream regulators and effectors within this signaling axis is critical for interrupting metastatic cascades and improving patient prognosis.

In recent years, accumulating evidence has demonstrated that long non-coding RNAs (lncRNAs) play pivotal regulatory roles in the development and progression of various cancers, including CRC [19,20]. Small nucleolar RNA host gene 5 (SNHG5), a lncRNA located at chromosome 6q14.3 with an approximate length of ∼2700 nucleotides, has been increasingly recognized as an oncogenic driver across multiple malignancies [21]. Aberrant upregulation of SNHG5 has been observed in diverse tumor types and is frequently associated with advanced disease stage and poor prognosis [22]. In CRC, elevated SNHG5 expression correlates with later TNM stage and reduced overall survival [23]. Functional studies further support its oncogenic role. As reported by Damas et al., SNHG5 is predominantly localized in the cytoplasm of CRC cells, where it binds to the double-stranded RNA-binding protein STAU1, thereby blocking STAU1-mediated mRNA degradation and stabilizing multiple pro-survival transcripts [24]. Knockdown of SNHG5 leads to cell cycle arrest, increased apoptosis, and reduced tumor growth in xenograft models. Additionally, SNHG5 functions as a competing endogenous RNA (ceRNA). It sponges miR-132-3p, leading to derepression of the transcription factor CREB5, which in turn promotes CRC cell proliferation, migration, and invasion [25]. CREB5 acts upstream of EMT regulators and enhances mesenchymal marker expression such as Vimentin, contributing to increased cell motility [26]. Beyond CRC, SNHG5 has been implicated in other malignancies through diverse mechanisms. In hepatocellular carcinoma (HCC), it interacts with UPF1 and activates Wnt/β-catenin signaling, maintaining stemness and promoting proliferation [27]. In non-small cell lung cancer, SNHG5 sponges miR-181c-5p to upregulate CBX4, thereby activating the NF-κB pathway and facilitating tumor progression [28]. Collectively, these findings highlight SNHG5 as a pro-metastatic lncRNA with pleiotropic functions, acting via ceRNA networks, inhibition of mRNA decay, and direct protein interactions. However, emerging data also suggest context-dependent duality. For instance, in esophageal carcinoma, SNHG5 was reported to suppress EMT and cell migration by interacting with MTA2 and promoting its ubiquitin-mediated degradation [29]. This heterogeneity underscores the need to elucidate the tissue-specific regulatory mechanisms of SNHG5, particularly its functional role in distant metastasis of CRC, which remains insufficiently characterized.

Mechanistically, SNHG5 promotes tumor progression through multiple regulatory pathways. (1) ceRNA axis: SNHG5 functions as a molecular sponge for tumor-suppressive microRNAs (miRNAs), thereby derepressing oncogenic downstream targets. In CRC, SNHG5 binds and sequesters miR-132-3p, leading to the upregulation of the transcription factor CREB5 and enhancement of cell proliferation, migration, and metastatic potential [30]. Similar ceRNA networks involving SNHG5 have been reported in other malignancies, including the SNHG5–miR-154-5p–PCNA axis in breast cancer [31] and the SNHG5–miR-26a-5p–GSK3β axis in HCC [32]. In particular, SNHG5 upregulates GSK3β by competitively binding miR-26a-5p in HCC, thereby aberrantly activating Wnt/β-catenin signaling and inducing EMT, which contributes to invasion and metastasis [32]. (2) RNA–protein interactions: SNHG5 also exerts its oncogenic effects by binding directly to regulatory proteins and modulating mRNA homeostasis. In CRC, SNHG5 interacts with the double-stranded RNA-binding protein Staufen1 (STAU1), preventing STAU1-mediated degradation of specific mRNAs and thereby stabilizing transcripts critical for cancer cell survival [24]. Similarly, in liver cancer, SNHG5 binds to UPF1, an RNA surveillance factor, impairing its function and resulting in the upregulation of Wnt/β-catenin pathway components, which supports stemness and self-renewal of cancer cells [27]. Through these complementary mechanisms, SNHG5 facilitates sustained activation of tumor-promoting signaling cascades, thereby enhancing proliferation, invasiveness, and resistance to apoptosis. However, it is important to note that SNHG5 exhibits context-dependent roles across cancer types. In gastric cancer, for instance, contrasting results have been reported. Zhao et al. found that elevated SNHG5 expression sequestered MTA2 protein in the cytoplasm, suppressing proliferation, migration, and invasion while promoting apoptosis [33]. Conversely, Li et al. demonstrated that SNHG5 was upregulated under cisplatin exposure, where it inhibited apoptosis and promoted chemoresistance [34]. Such functional discrepancies may reflect variations in tumor microenvironment, genomic context, or miRNA/protein interaction networks. Although SNHG5 has been widely recognized as a metastasis-promoting lncRNA, the precise molecular mechanisms by which it contributes to CRC—particularly in liver metastasis—remain incompletely understood. Whether SNHG5 regulates distant metastasis through specific ceRNA axes, protein interactions, or crosstalk with metabolic and immune pathways warrants further investigation. Elucidating these mechanisms will be essential for evaluating SNHG5 as a potential therapeutic target and advancing strategies to combat metastatic CRC.

Another significant research focus is the emerging role of G protein signaling pathways in the progression of CRC. Heterotrimeric G proteins, composed of Gα, Gβ, and Gγ subunits, mediate downstream signaling of G protein-coupled receptors (GPCRs), and regulate key cellular processes such as proliferation and migration [35]. The GNB2 gene encodes the β2 subunit of the G protein (Gβ2), a critical component of the G protein complex. Upon GPCR activation by ligands, Gβ2 dissociates from Gα along with Gγ, triggering multiple signaling cascades including PI3K/AKT and MAPK pathways, which collectively influence cellular behavior [36]. Recent multi-omics analyses have revealed aberrant overexpression of GNB2 across a range of human cancers. For instance, a pan-cancer study by Pashirzad et al. demonstrated that GNB2 was significantly upregulated in 23 tumor types compared to corresponding normal tissues [37]. More importantly, high GNB2 expression was strongly associated with poor patient prognosis. In both hepatocellular carcinoma and rectal adenocarcinoma, elevated GNB2 levels correlated with reduced overall survival [37]. These findings suggest that GNB2 may contribute to the malignant progression of gastrointestinal tumors and holds potential as a prognostic biomarker. Functional studies further support the oncogenic role of GNB2. Lane et al. reported that activating mutations in GNB2 drive abnormal cell proliferation and transformation through sustained activation of canonical G protein signaling pathways [35]. In hematologic malignancy models, introduction of mutant GNB2 conferred cytokine-independent growth advantages and resistance to multiple kinase inhibitors [35]. Although direct evidence for GNB2 function in solid tumors such as CRC remains limited, emerging clues point to its involvement in invasive progression. A gene expression meta-analysis involving 651 stage II CRC patients identified GNB2 as a central “hub” gene within a recurrence-associated network [38]. Bioinformatic screening of hundreds of metastasis-related differentially expressed genes revealed extensive interactions between GNB2 and key metastasis-associated proteins, highlighting its potential contribution to CRC recurrence [38]. Despite this growing evidence, the precise mechanisms by which GNB2 promotes malignant progression in CRC remain poorly defined. For example, how does GNB2 overexpression affect cellular invasiveness and motility? Through which G protein-mediated pathways—such as sphingolipid signaling or inflammatory cytokine signaling—might it modulate tumor–microenvironment interactions or immune evasion? These critical questions remain to be fully addressed.

Collectively, SNHG5, a long non-coding RNA, and GNB2, a critical subunit of the heterotrimeric G protein complex, have both been implicated as oncogenic factors in the progression of CRC. However, whether a functional regulatory interaction exists between these two molecules remains unclear. In particular, the mechanistic role of the putative SNHG5–GNB2 axis in CRC metastasis, especially liver metastasis, has yet to be systematically elucidated. Previous studies have primarily focused on the role of SNHG5 in promoting tumor cell proliferation and local invasion, whereas its potential involvement in key steps of distant metastasis—such as pre-metastatic niche formation, circulatory survival, and hepatic colonization—remains poorly understood. Meanwhile, although GNB2 has been found to be aberrantly upregulated in various malignancies and is closely associated with poor prognosis, its functional role and downstream signaling pathways in CRC metastasis lack direct experimental validation. Importantly, it remains to be determined whether SNHG5 can regulate the expression or activity of GNB2—either directly or indirectly—to co-drive metastatic phenotypes such as enhanced migration, invasion, survival, and immune evasion in CRC cells. Moreover, whether the SNHG5–GNB2 axis functionally intersects with classical signaling cascades, such as the Wnt/β-catenin pathway, to promote EMT and shape a pro-metastatic microenvironment in the liver, also warrants further investigation. A comprehensive understanding of the mechanistic function of the SNHG5–GNB2 axis in CRC liver metastasis may not only deepen insights into the regulatory networks underlying metastasis, but also reveal novel therapeutic targets and inform the development of personalized combinatorial intervention strategies.

## 2 Materials and methods

### 2.1 Cell line preparation and RNA sequencing

To obtain colorectal cancer cells with distinct metastatic phenotypes, we established two MC38-derived murine CRC sublines (designated as F0 and F3) through serial in vivo selection based on liver metastasis capacity in C57BL/6 mice. The parental F0 line represents low-metastatic potential, while the F3 line was derived after three rounds of splenic injection and liver metastasis isolation, resulting in a high-metastatic phenotype. Total RNA from three biological replicates of each subline was extracted using TRIzol reagent (Takara, Japan) according to the manufacturer's instructions. RNA quality was assessed using a NanoDrop 2000 (Thermo Fisher Scientific, USA) and Agilent 2100 Bioanalyzer. RNA-seq libraries were constructed from ribosomal RNA-depleted total RNA and sequenced on the Illumina NovaSeq 6000 platform (paired-end, 150 bp). Clean reads were generated by Skewer (v0.2.2) for adapter trimming and quality filtering. Reads were then aligned to the mouse lncRNA reference (NONCODE v6.0), including both mature and precursor transcripts. Known lncRNAs were identified, and expression levels were calculated using CPM (counts per million) and normalized using the TMM (trimmed mean of M values) method.

### 2.2 Biotin-labeled RNA pull-down coupled with mass spectrometry

To identify Snhg5-interacting proteins, biotin-labeled RNA pull-down was performed followed by LC–MS/MS analysis. Full-length sense and antisense RNA probes corresponding to the mouse Snhg5-203 isoform were synthesized by in vitro transcription and biotinylated using Biotin-16-UTP (Beyotime). The labeled RNAs were incubated with streptavidin-conjugated magnetic beads (Pierce) and rotated at 4 °C for 1 h with cytoplasmic lysates prepared from MC38-F3 cells. After stringent washing, the RNA–protein complexes were eluted and resolved by SDS-PAGE. The gel bands were excised and subjected to in-gel trypsin digestion. Peptides were analyzed on an EASY-nanoLC 1000 system coupled to a timsTOF Pro2 mass spectrometer (Bruker). MS/MS data were searched against the UniProt mouse database using PEAKS Studio X+ with the following parameters: trypsin specificity, precursor tolerance of 7 ppm, fragment tolerance of 0.02 Da, fixed modification of carbamidomethylation (C), and variable modifications including oxidation (M), deamidation (NQ), and protein N-terminal acetylation. Proteins with at least one unique peptide and a −10lgP score ≥15 were considered reliably identified. Differentially enriched proteins between sense and antisense groups were analyzed based on spectral count. Enrichment analysis for GO terms and KEGG pathways was performed using DAVID and KOBAS. Protein–protein interaction networks were constructed using STRING (v11.5) and visualized via Cytoscape.

### 2.3 In vivo metastasis model and bioluminescence imaging

Six-week-old male BALB/c nude mice and C57BL/6 mice (18–20 g) were obtained from Jiangsu Jicui Yaokang Biological Technology Co., Ltd. (SCXK [Su] 2023-0004) and maintained under specific pathogen-free (SPF) conditions. All procedures were approved by the Institutional Animal Care and Use Committee of Tongji Hospital (Approval No. T1-202405039), in accordance with NIH guidelines. To establish a liver metastasis model, 1 × 10^6^ MC38 murine colon carcinoma cells stably expressing firefly luciferase were suspended in 100 μL PBS and injected into the spleen under anesthesia (1.5 % pentobarbital sodium, 50 mg/kg, intraperitoneally). A small abdominal incision was made to expose the spleen. Following injection, the site was compressed with a sterile cotton swab for 1 min to achieve hemostasis and minimize cell leakage. The spleen was repositioned, and the incision was closed with 4-0 non-absorbable sutures. Mice were monitored daily for clinical signs and body weight. On day 30 post-injection, tumor burden was assessed by in vivo bioluminescence imaging using the Lago X system (Spectral Instruments Imaging). Mice were anesthetized and administered D-luciferin (150 mg/kg, intraperitoneally). After 10 min, images were acquired with an exposure time of 30 s. Regions of interest (ROIs) encompassing the liver were defined, and photon flux (photons/sec) was quantified using the system's analysis software. Mice were euthanized immediately after imaging. Livers were harvested, fixed in 4 % paraformaldehyde, and embedded for histological analysis. Metastatic burden was quantified by calculating the metastatic lesion area relative to the total liver area using ImageJ software (NIH, USA).

### 2.4 Public data collection and bioinformatic analysis

Transcriptomic and clinical data for CRC were obtained from TCGA-COAD via the UCSC Xena browser (https://xenabrowser.net). GNB2 expression profiles and associated survival information were extracted for downstream analysis. Gene expression datasets GSE37182, GSE35982, and GSE49355 were downloaded from the GEO database (https://www.ncbi.nlm.nih.gov/geo) to assess GNB2 mRNA levels in CRC tissues, adjacent normal tissues, and liver metastases. SNHG5 expression was analyzed using GSE37182 and GSE71187. Group comparisons were performed using the Wilcoxon rank-sum test. Kaplan–Meier survival analysis was conducted using both TCGA-COAD data and an external rectal adenocarcinoma dataset via the Kaplan–Meier Plotter (https://kmplot.com). Patients were stratified into high and low expression groups by median cutoff. Survival curves were compared using the log-rank test, and hazard ratios were calculated. Immunohistochemical staining images and scoring data for GNB2 in normal colon and CRC tissues were obtained from the Human Protein Atlas (HPA) using antibody HPA040736. Quantitative comparison of GNB2-positive staining was performed between groups.

### 2.5 RNA fluorescence in situ hybridization (RNA-FISH)

Formalin-fixed, paraffin-embedded (FFPE) liver tissue sections containing metastatic lesions from F0 and F3 groups were subjected to RNA-FISH using a Cy3-labeled Snhg5-specific probe (Servicebio, China). Briefly, liver tissues were fixed in in situ hybridization fixative for at least 12 h at 4 °C, followed by dehydration in graded ethanol, xylene clearing, and paraffin embedding. Serial 4-μm sections were cut, mounted, and baked at 62 °C for 2 h. After dewaxing and rehydration, antigen retrieval was performed, followed by proteinase K digestion (20 μg/mL, 40 °C). Slides were then prehybridized at 40 °C for 1 h, and hybridized overnight with the Cy3-labeled Snhg5 probe in a humidified chamber. Post-hybridization washes were carried out sequentially in 2 × SSC (10 min at 40 °C), 1 × SSC (5 min × 2 at 40 °C), and 0.5 × SSC (10 min at room temperature). Nuclei were counterstained with DAPI, and slides were mounted with antifade reagent. Images were acquired using a Nikon Eclipse CI upright fluorescence microscope equipped with a DS-U3 imaging system. U6 served as the nuclear control. Signal intensity was quantified using ImageJ software and statistically compared between F0- and F3-derived liver metastasis groups.

### 2.6 RNA immunoprecipitation (RIP) assay

RNA immunoprecipitation (RIP) was performed using the Magna RIP™ RNA-Binding Protein Immunoprecipitation Kit (Millipore, Cat. No. 17–700) according to the manufacturer's instructions. MC38-F3 colorectal cancer cells (1 × 10^7^) were harvested and lysed in RIP lysis buffer supplemented with RNase inhibitor (40 U/mL; Thermo Fisher) and protease inhibitor cocktail (Roche) on ice for 5 min. After clarification by centrifugation at 14,000×*g* for 10 min at 4 °C, the supernatants were collected for subsequent immunoprecipitation. Magnetic protein A/G beads were incubated with 5 μg of anti-GNB2 antibody (Abcam, ab108504) or normal rabbit IgG (Millipore) at room temperature for 30 min with gentle rotation. The antibody-conjugated beads were then incubated with the clarified lysates at 4 °C overnight with continuous mixing. The next day, the beads were sequentially washed with high-stringency buffers provided in the kit. Bead-bound complexes were digested with proteinase K at 55 °C for 30 min. Total RNA was extracted using phenol-chloroform and precipitated with ethanol. Recovered RNA was reverse transcribed using the PrimeScript™ RT Reagent Kit (Takara, Japan), and enrichment of SNHG5 in the RIP fraction was measured by SYBR-based qRT-PCR. Relative enrichment was normalized to input RNA and expressed as fold enrichment over the IgG negative control. The biotin-labeled sense and antisense RNA probes targeting mouse Snhg5 were synthesized via in vitro transcription using primers listed in Supplementary Table S1.

### 2.7 Biotin-labeled RNA pull-down assay

Full-length sense and antisense Snhg5 transcripts were in vitro transcribed using T7 RNA Polymerase (Beyotime, R7012M) and Biotin-16-UTP (Beyotime, D7336) and purified with RNA Clean & Concentrator-25 (Zymo). Biotinylated RNA (3 μg) was denatured at 90 °C for 2 min, snap-cooled, and structured at room temperature for 20 min. Pull-down was conducted using the Bersin RNA Pulldown Kit (Bes5102) according to the manufacturer's protocol. RNA was incubated with streptavidin magnetic beads (40 μL) at 25 °C for 30 min, then with cytoplasmic lysates from MC38-F3 cells (1 × 10^7^) in RIP buffer containing RNase inhibitor (Thermo, 10777019) and Poly(dI·dC). After 2 h incubation at 25 °C, beads were washed with NT2 buffer and eluted in SDS loading buffer. Pulled-down proteins were analyzed by Western blot using anti-GNB2 (Abcam, ab108504). Antisense and bead-only controls were included.

### 2.8 Cell culture

The murine colorectal carcinoma cell line MC38 was obtained from the American Type Culture Collection (ATCC, USA) and cultured in high-glucose DMEM (Gibco, USA) supplemented with 10 % fetal bovine serum (FBS), 100 U/mL penicillin, and 100 μg/mL streptomycin. Cells were maintained at 37 °C in a humidified incubator containing 5 % CO_2_. For in vivo imaging studies, MC38 cells were transduced with a lentiviral vector encoding firefly luciferase. Stable clones were selected and expanded under standard culture conditions.

### 2.9 Lentiviral vector construction and transduction

Lentiviral vectors expressing short hairpin RNAs (shRNAs) targeting murine Snhg5 and Gnb2 were custom-designed and synthesized by Obio Technology Co., Ltd. (Shanghai, China). For each gene, three independent shRNA sequences were cloned into the pCLenti-U6-shRNA-CMV-EGFP-F2A-BSR-WPRE vector backbone (plasmid ID: GL223). The target sequences and corresponding genomic loci (RefSeq) are listed in Supplementary Table S2. A non-targeting control construct (pCLenti-U6-shRNA-NC, plasmid ID: Y15236) was used as a negative control. Lentiviral particles were produced using a third-generation packaging system and concentrated to a functional titer of ≥1.0 × 10^8^ TU/mL. For transduction, target cells were seeded at 40–60 % confluency and infected at a multiplicity of infection (MOI) of 10 in the presence of 8 μg/mL polybrene (Sigma-Aldrich, USA). Following 24 h of incubation, the culture medium was replaced, and transduced cells were selected using 10 μg/mL blasticidin (InvivoGen) for 72 h prior to downstream analyses.

### 2.10 Cell wound healing assay

Cells (1 × 10^6^ per well) were seeded into six-well plates and cultured for 24 h until reaching 80–90 % confluence. A linear scratch was created across the cell monolayer using a sterile 10 μL pipette tip. After washing with PBS to remove detached cells, the cultures were maintained in DMEM supplemented with 2 % fetal bovine serum. Wound closure was monitored at 0 and 48 h using phase-contrast microscopy. Images were acquired at three randomly selected fields ( × 200 magnification) per well. The migration distance was measured as the gap width reduction over time, and cell motility was quantified accordingly.

### 2.11 Colony formation assay

Cells were seeded into six-well plates at a density of 500 cells per well and cultured for 10–14 days until visible colonies formed. Colonies were fixed with methanol for 15 min, stained with 1 % crystal violet for 20 min at room temperature, and rinsed gently with PBS to remove excess dye. Plates were air-dried, and colonies containing more than 50 cells were manually counted under a light microscope. Triplicate wells were analyzed per group.

### 2.12 Transwell migration and invasion assay

Cell migration and invasion were assessed using Transwell chambers with 8 μm pore polycarbonate membranes (#3422, Corning, USA). For the invasion assay, the upper surface of the membrane was pre-coated with 50 μL of Matrigel (BD Biosciences, USA) and incubated at 37 °C for 1 h. For migration assays, no Matrigel coating was applied. Cells (2 × 10^4^ per well) suspended in 200 μL of serum-free DMEM were seeded into the upper chamber, and 500 μL of DMEM containing 10 % FBS was added to the lower chamber as a chemoattractant. After incubation at 37 °C for 18 h, non-migrated cells were gently removed from the upper surface of the membrane using a cotton swab. Cells on the underside of the membrane were fixed with 4 % paraformaldehyde for 15 min, stained with 1 % crystal violet for 20 min, and washed with PBS. Stained cells were imaged using an inverted microscope (SDPTOP, China), and the number of migrated or invaded cells was counted in five randomly selected fields per insert at 200 × magnification.

### 2.13 CCK-8 cell proliferation assay

Cell proliferation was assessed using the Cell Counting Kit-8 (CCK-8; HY-K0301, MedChemExpress, China) following the manufacturer's instructions. Cells were seeded into 96-well plates at a density of 3 × 10^3^ cells per well in 200 μL of complete medium and incubated under standard culture conditions. At 24, 48, 72, and 96 h, the medium was removed and replaced with 100 μL fresh medium containing 10 μL of CCK-8 reagent. After a 2-h incubation at 37 °C in the dark, absorbance at 450 nm was measured using a microplate reader (ELx800, BioTek Instruments, USA). Each experiment was performed in triplicate.

### 2.14 EdU proliferation assay

Cell proliferation was evaluated using the Cell-Light™ EdU Apollo 567 In Vitro Kit (Cat. No. C10310-1; RiboBio Technology Co., Ltd., Guangzhou, China). Following transfection, cells were seeded into 96-well plates at a density of 5 × 10^3^ cells per well and incubated overnight for adherence. On the next day, cells were incubated with EdU labeling medium (final concentration: 50 μM) for 2 h at 37 °C. Subsequently, cells were fixed in 4 % paraformaldehyde, neutralized with 2 mg/mL glycine, and permeabilized using 0.5 % Triton X-100 for 30 min. After washing with PBS, the cells were stained with Apollo 567 fluorescent reaction solution and counterstained with Hoechst 33342. Fluorescence images were captured using a Leica DMI3000B inverted fluorescence microscope.

### 2.15 Cell cycle analysis

Cell cycle distribution was determined by propidium iodide (PI) staining followed by flow cytometry. Transfected cells were fixed in 70 % ice-cold ethanol at 4 °C for at least 2 h. After washing, cells were incubated with 100 μL PI staining solution (100 μg/mL; Beyotime, China) at 37 °C for 30 min in the dark. Samples were analyzed using a CytoFLEX flow cytometer (Beckman Coulter, USA), and cell cycle phases were quantified with ModFit LT 3.2 software (Verity Software House, USA).

### 2.16 Annexin V–FITC/PI apoptosis assay

Apoptosis was assessed using the Annexin V–FITC/PI Apoptosis Detection Kit (BD Pharmingen, Cat. No. 556547, USA) in accordance with the manufacturer's instructions. Briefly, 1 × 10^5^ cells from each treatment group were collected by trypsinization (excluding EDTA), washed twice with cold 1 × Binding Buffer, and resuspended in 200 μL of the same buffer. Subsequently, 5 μL of Annexin V–FITC and 5 μL of propidium iodide (PI) were added to the cell suspension. Samples were incubated for 15 min at room temperature in the dark. After staining, 300 μL of 1 × Binding Buffer was added to bring the total volume to 500 μL. Samples were gently mixed and analyzed within 1 h using a CytoFLEX flow cytometer (Beckman Coulter, USA). A minimum of 10,000 events per sample were collected. Data were processed using FlowJo software (version 10.9.0; BD Biosciences), and apoptotic cells were classified as follows: early apoptosis (Annexin V^+^/PI^−^), late apoptosis or secondary necrosis (Annexin V^+^/PI^+^), viable cells (Annexin V^−^/PI^−^), and necrotic or mechanically damaged cells (Annexin V^−^/PI^+^).

### 2.17 Subcellular fractionation

Nuclear and cytoplasmic RNA fractions were isolated using the Nuclear and Cytoplasmic Extraction Kit (E101, Vazyme Biotech, China) in accordance with the manufacturer's instructions. Briefly, cells were harvested, lysed sequentially with cytoplasmic and nuclear extraction buffers on ice, and centrifuged to separate subcellular compartments. The cytoplasmic and nuclear fractions were collected and subjected to RNA extraction using TRIzol reagent (Takara, Japan). Isolated RNA was either immediately used or stored at −80 °C for downstream analysis.

### 2.18 Western blot analysis

Total cellular proteins were extracted using RIPA lysis buffer (Beyotime, China) supplemented with 1 % phenylmethylsulfonyl fluoride (PMSF). Protein concentrations were determined using a bicinchoninic acid (BCA) assay kit (BOSTER, Wuhan, China), following the manufacturer's instructions. Equal amounts of protein (30–50 μg per lane) were resolved by SDS-PAGE on 10 % polyacrylamide gels, with a stacking voltage of 70 V and resolving voltage of 100 V. Proteins were then transferred onto PVDF membranes (Millipore, USA) at 200 mA for 2 h in transfer buffer containing 20 % methanol. Membranes were blocked in 5 % non-fat dry milk diluted in TBST (Tris-buffered saline containing 0.1 % Tween-20) for 1 h at room temperature and incubated overnight at 4 °C with the following primary antibodies: GNB2 (1:1000; rabbit monoclonal, clone EP3262Y, Abcam, ab108504), E-cadherin (1:1000; rabbit monoclonal, CST, #14472), N-cadherin (1:1000; rabbit monoclonal, CST, #13116), Vimentin (1:1000; rabbit polyclonal, Proteintech, 10366-1-AP), Slug (1:1000; rabbit monoclonal, CST, #9585) and GAPDH (1:5000; mouse monoclonal, Proteintech, 60004-1-Ig) as a loading control. After washing with TBST, membranes were incubated for 1 h at room temperature with horseradish peroxidase (HRP)-conjugated goat anti-rabbit or goat anti-mouse secondary antibodies (1:10,000; Cell Signaling Technology). Protein bands were visualized using an enhanced chemiluminescence (ECL) detection kit (Thermo Fisher Scientific, USA) and imaged with a G:BOX Chemi X imaging system (Syngene, UK). Densitometric quantification was performed using ImageJ software (NIH, USA).

### 2.19 Cycloheximide chase assay for GNB2 protein stability

GNB2 protein stability was assessed by cycloheximide (CHX) chase. MC38-F3 cells stably transduced with sh-NC, sh-Snhg5 or sh-Snhg5 plus Snhg5 re-expression were plated in six-well plates and cultured to approximately 80 % confluence. Protein synthesis was blocked by adding CHX (100 μg/mL; C7698, Sigma-Aldrich/Merck) to pre-warmed complete DMEM; parallel cultures without CHX (CHX-free) served as negative controls. Cells were harvested at 0, 4 and 8 h, washed with cold PBS and lysed in RIPA buffer containing protease inhibitors as for Western blotting. Equal amounts of protein from each time point were resolved by SDS-PAGE and immunoblotted for GNB2 and GAPDH. Band intensities were quantified with ImageJ, and GNB2 signals were normalized to GAPDH and then to the corresponding 0 h value to obtain the fraction of protein remaining. Protein decay curves and half-life values were calculated by fitting a one-phase exponential decay model in GraphPad Prism. Each experiment was performed in at least three independent biological replicates.

### 2.20 Quantitative real-time PCR

Total RNA was extracted from cultured cells and tissue samples using TRIzol reagent (Takara, Japan). RNA concentration and purity were determined using a NanoDrop 2000 spectrophotometer (Thermo Fisher Scientific, USA). First-strand cDNA was synthesized using a S1000 thermal cycler (Bio-Rad, USA). Quantitative PCR was performed using a 2 × SYBR Green qPCR Master Mix (Low ROX; Bimake, China) on a Quantagene q225 real-time PCR system (KUBO Technology Co., Ltd., Beijing, China), with gene-specific primers synthesized by TSINGKE (Beijing, China). The thermal cycling protocol consisted of an initial denaturation at 95 °C for 30 s, followed by 40 cycles of denaturation at 95 °C for 15 s, annealing at 60 °C for 30 s, and extension at 72 °C for 30 s. Melt curve analysis was performed with 95 °C for 15 s, 60 °C for 60 s, and 95 °C for 15 s. Relative gene expression levels were calculated using the 2^–ΔΔCt method.

### 2.21 Actinomycin D chase assay for Gnb2 mRNA stability

To examine whether Snhg5 regulates Gnb2 mRNA stability, an actinomycin D (ActD) chase assay was performed. MC38-F3 cells stably expressing shCtrl or shSnhg5 were seeded in six-well plates (2 × 10^5 cells per well) and grown in complete DMEM to 70–80 % confluence. Transcription was then inhibited by adding ActD (5 μg/mL; A9415, Sigma-Aldrich/Merck) to the culture medium. Cells were collected at 0, 2, 4, 6 and 8 h after treatment, washed with ice-cold PBS, and total RNA was extracted using TRIzol (Takara, Japan). Gnb2 and Gapdh mRNA levels were quantified by SYBR Green–based qRT-PCR as described above. For each condition, Gnb2 expression at each time point was normalized to Gapdh and expressed as a fraction of the 0 h value (set to 1.0). mRNA decay curves and half-life estimates were obtained by nonlinear regression using a one-phase exponential decay model in GraphPad Prism. All experiments were independently repeated at least three times.

### 2.22 Hematoxylin and eosin (H&E) staining

Liver tissues from nude mice were fixed in 4 % paraformaldehyde, embedded in paraffin, and sectioned at a thickness of 5 μm. Sections were deparaffinized in xylene (two changes) and rehydrated through a graded ethanol series (100 %, 95 %, 85 %, and 75 %, each for 5 min), followed by rinsing in distilled water. Slides were stained with hematoxylin and eosin according to standard protocols. After staining, sections were dehydrated with 95 % ethanol and absolute ethanol (two changes), cleared in xylene (two changes), and mounted using neutral balsam. Histological evaluation of liver metastatic lesions was performed using a light microscope.

### 2.23 Immunohistochemistry (IHC)

Immunohistochemistry was performed to assess protein expression in liver metastatic tissues derived from intrasplenic CRC mouse models. Paraffin-embedded liver sections (5 μm) were deparaffinized in xylene, rehydrated through a graded ethanol series, and subjected to endogenous peroxidase blocking with 3 % hydrogen peroxide for 10 min. Antigen retrieval was carried out in citrate buffer (pH 6.0) using microwave heating for 15 min. After cooling to room temperature, sections were blocked with 5 % goat serum for 30 min and incubated overnight at 4 °C with primary antibodies against Ki67 (1:200, Abcam, ab15580), β-catenin (1:100, CST, #8480), E-cadherin (1:100, CST, #3195), and Vimentin (1:200, Abcam, ab92547). HRP-conjugated secondary antibodies (1:200, Zsbio, China) were applied for 30 min at room temperature, and signals were developed with DAB. Slides were counterstained with hematoxylin, dehydrated, cleared, and mounted. Stained tissues were visualized using a Leica DMi8 microscope. Quantitative analysis of immunoreactivity was performed using Fiji (ImageJ-win64) by calculating integrated optical density (IOD) in five randomly selected high-power fields per sample.

### 2.24 Immunofluorescence staining

F0 and F3 colorectal cancer cells were seeded onto sterilized glass coverslips in 24-well plates and cultured until approximately 70–80 % confluence. Cells were fixed with 4 % paraformaldehyde (Solarbio, China) for 15 min at room temperature, followed by permeabilization with 0.3 % Triton X-100 (Sigma-Aldrich, USA) for 15 min. After blocking in 5 % bovine serum albumin (BSA; Sigma-Aldrich) for 1 h, cells were incubated overnight at 4 °C with mouse monoclonal anti-E-cadherin (1:200, ab76055, Abcam) and rabbit polyclonal anti-Vimentin (1:200, ab92547, Abcam) antibodies. The next day, cells were washed and incubated with Alexa Fluor 488-conjugated goat anti-mouse and Alexa Fluor 594-conjugated goat anti-rabbit secondary antibodies (1:500; Thermo Fisher Scientific) for 1 h at room temperature in the dark. Nuclei were counterstained with DAPI (1 μg/mL; Sigma-Aldrich) for 5 min. Coverslips were mounted using ProLong™ Gold Antifade Reagent (Invitrogen, USA), and images were acquired using a Leica DMI3000B inverted fluorescence microscope under identical exposure settings for all samples. Immunofluorescence was performed in at least three independent replicates, and representative images were selected for presentation.

### 2.25 Statistical analysis

All statistical analyses were performed using GraphPad Prism 9.0 (GraphPad Software), IBM SPSS Statistics 29.0.2 (IBM Corp.), and R 4.4.0 (R Foundation). Data are presented as mean ± SEM from ≥3 independent experiments unless otherwise noted. Normality was assessed with the Shapiro–Wilk test. Two-group comparisons were evaluated using unpaired two-tailed Student's t-test or Mann–Whitney *U* test, as appropriate. One-way ANOVA with Tukey's post-hoc test or Kruskal–Wallis test with Dunn's adjustment was used for comparisons among ≥3 groups. Two-way ANOVA with Bonferroni correction was applied for factorial/time-course experiments. Transcriptomic data (TCGA-COAD, GEO datasets) were analyzed using non-parametric tests (Wilcoxon or Kruskal–Wallis) and corrected for multiple testing with the Benjamini–Hochberg method (FDR <0.05). Kaplan–Meier survival was assessed using the log-rank test, with hazard ratios estimated via Cox regression. Correlations were analyzed using Spearman's rank test. Significance was defined as P < 0.05 (two-sided) unless otherwise specified.

## 3 Results

### 3.1 In vivo selection yields a highly metastatic MC38-derived colorectal cancer subline with enhanced liver colonization potential

**Fig. 1:**
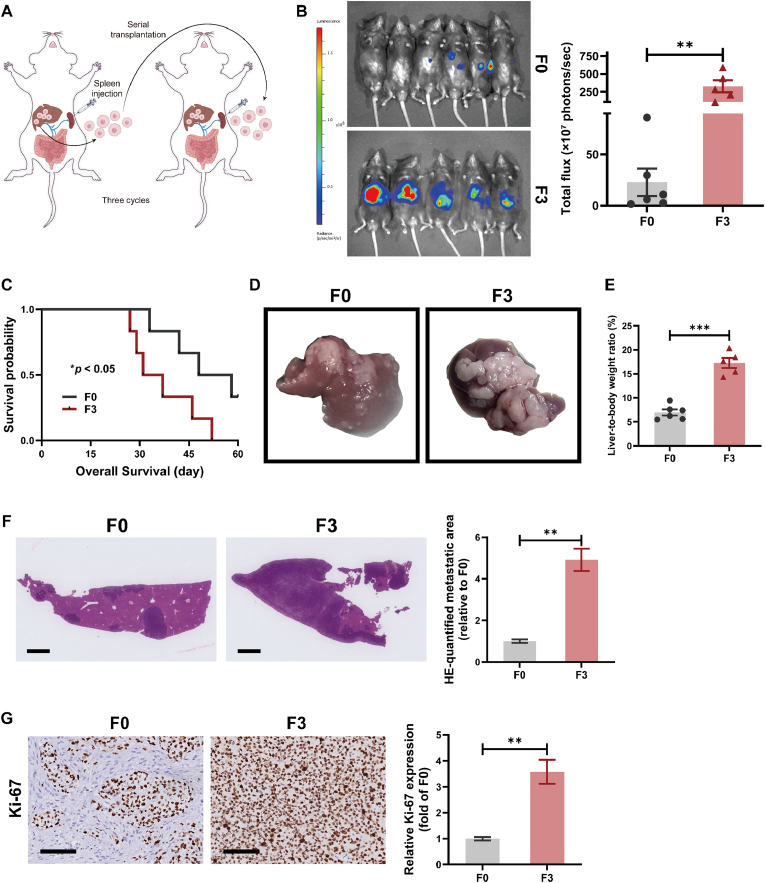
**Establishment of a highly metastatic MC38-derived CRC subline with enhanced liver colonization potential**. **(A)** Schematic illustration of the serial in vivo selection protocol used to generate a highly metastatic MC38-F3 subline. Parental MC38-F0 cells were intrasplenically injected into immunocompetent C57BL/6 mice. Hepatic metastatic lesions were harvested, expanded ex vivo, and re-implanted over three consecutive cycles to yield the F3 derivative. **(B)** Bioluminescence imaging performed on day 30 post-injection revealed markedly higher hepatic tumor burden in mice injected with MC38-F3 cells compared to MC38-F0 controls. Quantification of liver-associated photon flux confirmed significantly elevated metastatic load in the F3 group. **(C)** Kaplan–Meier survival analysis demonstrated significantly reduced overall survival in F3-bearing mice relative to F0 (log-rank test; P < 0.05). **(D)** Gross morphology of excised livers showed limited metastatic lesions in F0 mice and extensive nodular infiltration in F3 mice. **(E)** Liver-to-body weight (LW/BW) ratio was significantly increased in the F3 group, reflecting greater hepatic tumor burden. **(F)** Representative H&E-stained liver sections confirmed widespread metastatic infiltration in F3 mice. Quantification showed a significantly larger metastatic area relative to F0 (scale bars = 1 mm). **(G)** Immunohistochemical staining for Ki-67 demonstrated increased proliferative activity in F3-derived liver metastases compared to F0, as evidenced by a higher proportion of Ki-67–positive nuclei (scale bars = 100 μm). Data are presented as mean ± SEM. Statistical analyses were performed using unpaired two-tailed Student's *t*-test unless otherwise indicated. ∗*P* < 0.05, ∗∗*P* < 0.01, ∗∗∗*P* < 0.001.

To model colorectal cancer liver metastasis, we performed serial in vivo selection using the murine MC38 cell line. Low-metastatic MC38-F0 cells were intrasplenically injected into C57BL/6 mice, and hepatic metastatic lesions were harvested and re-implanted for three successive rounds to establish a highly metastatic subline, MC38-F3 (Fig. 1A). Bioluminescence imaging on day 30 post-injection revealed markedly increased hepatic tumor burden in F3-injected mice compared to the F0 group, as evidenced by elevated liver-associated photon flux (Fig. 1B). Kaplan–Meier survival analysis demonstrated significantly shorter overall survival in the F3 group (log-rank test; P < 0.05; Fig. 1C). Gross examination showed extensive, coalescent liver metastases in F3 mice, while only minimal lesions were observed in the F0 group (Fig. 1D). Quantification of the liver-to-body weight (LW/BW) ratio further confirmed a significantly higher tumor burden in the F3 group (Fig. 1E). Histological analysis by H&E staining revealed widespread metastatic infiltration in F3 livers, with a significantly larger metastatic area compared to F0 (Fig. 1F). Moreover, immunohistochemical staining for Ki-67 showed an increased proportion of proliferative tumor cells in F3-derived liver lesions (Fig. 1G). Collectively, these findings establish the MC38-F3 subline as a robust, liver-tropic model of colorectal cancer metastasis, with enhanced proliferative and colonization capacity in vivo.

### 3.2 F3 CRC cells exhibit enhanced proliferative, migratory, and invasive capacities with mesenchymal features

**Fig. 2:**
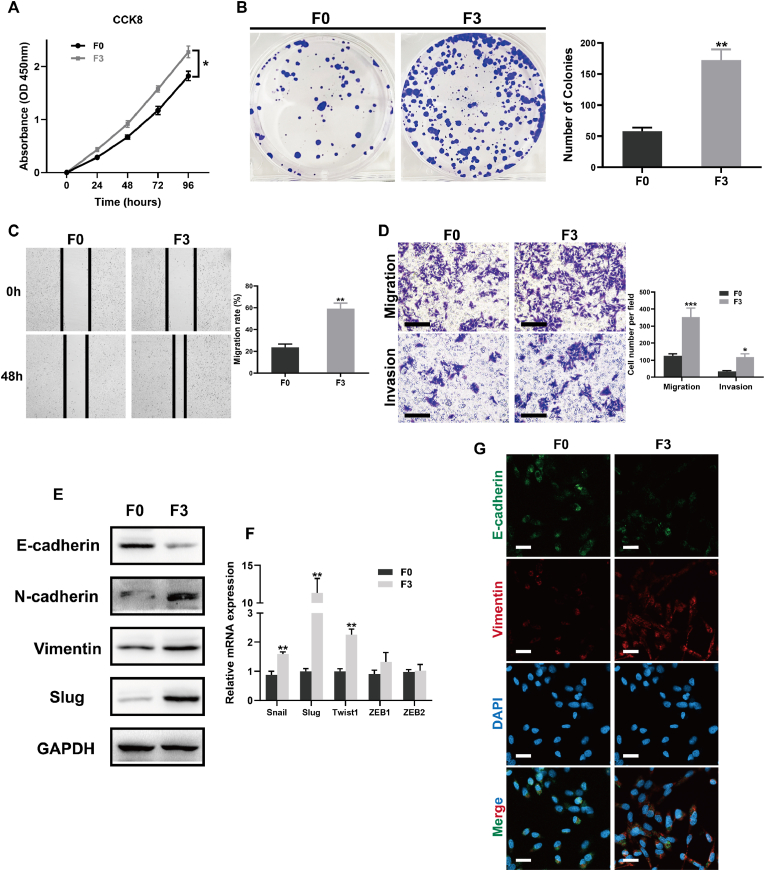
**Highly metastatic MC38-derived CRC cells exhibit enhanced proliferative, migratory, and invasive properties with mesenchymal characteristics**. **(A)** CCK-8 assay demonstrated significantly higher proliferative capacity in the highly metastatic MC38-F3 subline compared to the parental low-metastatic MC38-F0 cells over a 96-h time course. **(B)** Representative colony formation images and quantification revealed increased clonogenicity in the MC38-F3 subline. **(C)** Wound healing assays indicated enhanced migratory ability in MC38-F3 cells at 48 h post-scratch. **(D)** Transwell migration and Matrigel-coated invasion assays showed that MC38-F3 cells exhibited significantly increased motility and invasiveness. Scale bars = 100 μm. **(E)** Western blot analysis revealed downregulation of the epithelial marker E-cadherin and upregulation of mesenchymal markers N-cadherin, Vimentin, and Slug in MC38-F3 cells. **(F)** RT-qPCR analysis confirmed significant upregulation of EMT-associated transcription factors (Snail, Slug, Twist1, ZEB1, ZEB2) in MC38-F3 cells. **(G)** Immunofluorescence staining corroborated the EMT phenotype, showing reduced E-cadherin and increased Vimentin expression in MC38-F3 cells. Nuclei were counterstained with DAPI. Scale bars = 20 μm. Data are presented as mean ± SEM from at least three independent experiments. ∗*P* < 0.05, ∗∗*P* < 0.01, ∗∗∗*P* < 0.001 (Student's *t*-test).

To investigate phenotypic differences between the parental (F0) and liver metastasis-derived (F3) murine CRC cell lines, a series of in vitro functional assays were conducted. As shown in the CCK-8 assay, F3 cells displayed significantly greater proliferative activity than F0 cells over a 96-h period (p < 0.05; Fig. 2A). Similarly, colony formation assays revealed a substantial increase in the number of colonies formed by F3 cells, indicating enhanced clonogenic potential (p < 0.01; Fig. 2B). Wound healing assays demonstrated accelerated migratory ability in F3 cells, as evidenced by a significantly reduced wound area at 48 h compared to F0 cells (p < 0.01; Fig. 2C). Consistently, Transwell migration and invasion assays confirmed that F3 cells exhibited markedly increased motility and invasiveness relative to F0 cells (p < 0.001 and p < 0.05, respectively; Fig. 2D). To further examine whether these changes were associated with epithelial–mesenchymal transition (EMT), the expression of canonical EMT markers was assessed. Western blot analysis revealed reduced levels of E-cadherin, an epithelial marker, alongside upregulation of mesenchymal markers including N-cadherin, Vimentin, and Slug in F3 cells (Fig. 2E). Consistent with these findings, RT-qPCR showed significantly elevated mRNA levels of Snail, Slug, and Twist1 in F3 cells compared to F0 (p < 0.01; Fig. 2F). Immunofluorescence staining further corroborated these observations at the protein level, showing decreased E-cadherin and increased Vimentin expression in F3 cells (Fig. 2G). Taken together, these results demonstrate that F3 cells have acquired mesenchymal characteristics and possess enhanced proliferative, migratory, and invasive capabilities, supporting a more aggressive metastatic phenotype.

### 3.3 Snhg5 is upregulated in highly metastatic CRC cells

**Fig. 3:**
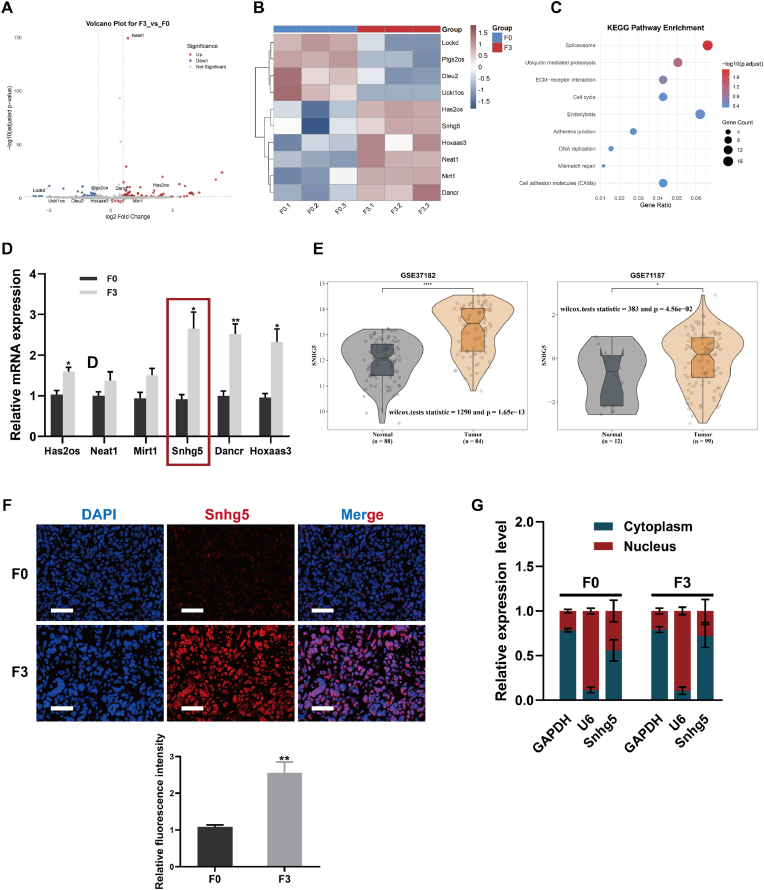
**Snhg5 is significantly upregulated in highly metastatic CRC cells and predominantly localized in the cytoplasm**. **(A)** Volcano plot of RNA-seq data showing differentially expressed lncRNAs between the low-metastatic parental MC38-F0 and the highly metastatic MC38-F3 CRC sublines; Snhg5 was among the most significantly upregulated transcripts in F3 cells. **(B)** Hierarchical clustering heatmap confirmed consistent overexpression of Snhg5 across biological replicates of the F3 subline. **(C)** KEGG enrichment analysis of Snhg5-correlated genes revealed significant association with tumor-related pathways, including cell cycle, ECM–receptor interaction, and adherens junctions. **(D)** RT-qPCR validation of selected lncRNAs identified Snhg5, Dncr, and Hoxaas3 as significantly upregulated in MC38-F3 cells relative to MC38-F0. **(E)** Violin plots derived from two independent GEO datasets (GSE37182 and GSE71187) showed that SNHG5 expression was markedly elevated in CRC tissues compared with matched normal controls. **(F)** RNA fluorescence in situ hybridization (RNA-FISH) of murine liver metastases revealed that Snhg5 signals were primarily cytoplasmic and significantly stronger in MC38-F3–derived lesions compared with MC38-F0. Scale bars = 50 μm. **(G)** Nuclear–cytoplasmic fractionation followed by RT-qPCR further confirmed predominant cytoplasmic localization of Snhg5 in both sublines, with a higher cytoplasmic ratio in the highly metastatic MC38-F3 cells. GAPDH and U6 served as cytoplasmic and nuclear controls, respectively. Data are presented as mean ± SEM. Statistical significance was determined using Student's *t*-test or Wilcoxon rank-sum test as appropriate. ∗*P* < 0.05, ∗∗*P* < 0.01.

To identify lncRNAs associated with metastatic potential, we performed transcriptomic profiling of colorectal cancer cell sublines with distinct liver metastatic capacities (F0 vs. F3). Volcano plot analysis revealed a set of significantly differentially expressed lncRNAs, among which Snhg5 was prominently upregulated in F3 cells (log_2_FC > 2, adjusted p < 0.001; Fig. 3A). Hierarchical clustering further confirmed the consistent upregulation of Snhg5 across all F3 replicates (Fig. 3B). To explore the biological relevance of these lncRNAs, KEGG pathway enrichment analysis was performed based on predicted target genes, revealing enrichment in tumor-related pathways such as cell cycle, ECM–receptor interaction, DNA replication, and adherens junctions (Fig. 3C). qRT-PCR validated the differential expression of candidate lncRNAs, confirming that Snhg5, along with Dncr and Hoxaas3, was significantly upregulated in F3 cells compared to F0 (p < 0.01; Fig. 3D). To evaluate clinical relevance, we analyzed two independent CRC cohorts from the GEO database (GSE37182 and GSE71187), where SNHG5 was consistently overexpressed in tumor tissues relative to normal controls (p < 0.001 and p = 0.0466, respectively; Fig. 3E). RNA-FISH analysis of metastatic liver tissues revealed that Snhg5 was predominantly localized in the cytoplasm, with significantly stronger fluorescence signals observed in the F3-derived liver metastases compared to F0 (p < 0.01; Fig. 3F). To confirm this localization, we performed nuclear–cytoplasmic fractionation followed by RT-qPCR. Consistent with the FISH results, Snhg5 expression was primarily distributed in the cytoplasmic compartment in both F0 and F3 cells, with a higher cytoplasmic ratio in the metastatic F3 group (Fig. 3G). Collectively, these findings identify Snhg5 as a consistently upregulated lncRNA in highly metastatic CRC cells, predominantly localized in the cytoplasm, suggesting a potential role in post-transcriptional regulation during CRC metastasis.

### 3.4 Snhg5 promotes proliferation and motility while inhibiting apoptosis in CRC cells

**Fig. 4:**
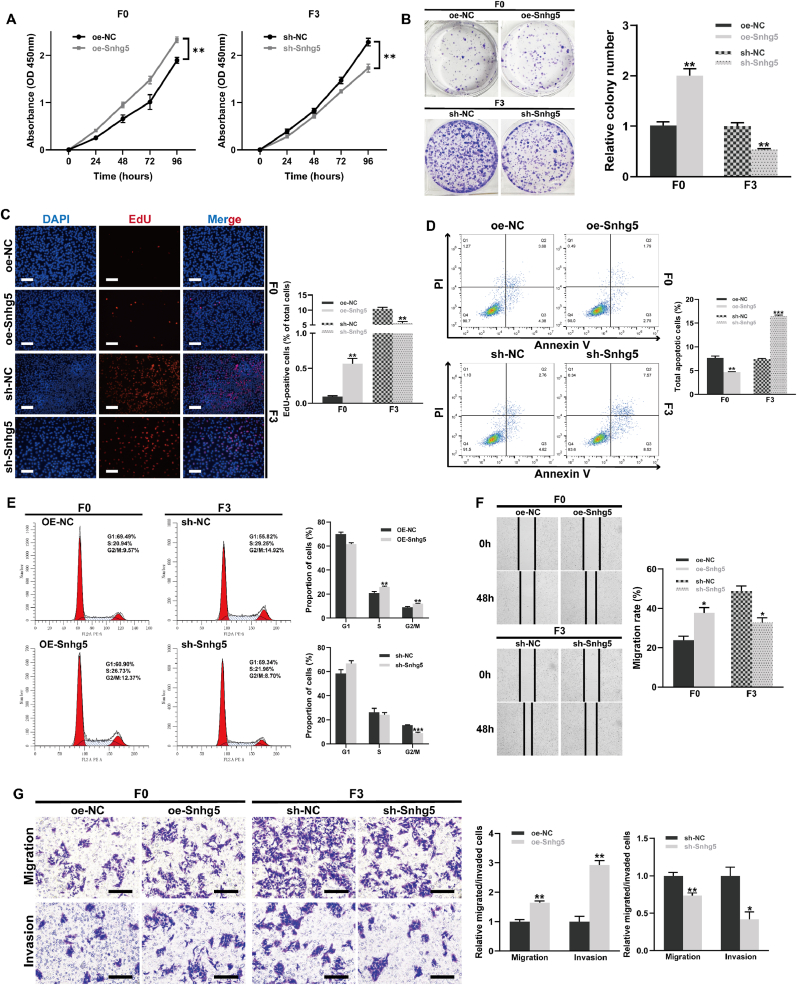
**Snhg5 promotes proliferation, inhibits apoptosis, and enhances motility in CRC cells**. **(A)** CCK-8 assays revealed that Snhg5 overexpression significantly promoted cell proliferation in low-metastatic MC38-F0 cells, while knockdown of Snhg5 suppressed proliferation in highly metastatic MC38-F3 cells over 96 h. **(B)** Colony formation assays showed increased clonogenic potential in Snhg5-overexpressing MC38-F0 cells and reduced colony formation following Snhg5 silencing in MC38-F3 cells. **(C)** EdU incorporation assays demonstrated elevated DNA synthesis in Snhg5-overexpressing cells and markedly reduced EdU-positive cell fractions upon Snhg5 knockdown. Scale bars = 100 μm. **(D)** Annexin V–FITC/PI staining followed by flow cytometry showed that Snhg5 overexpression reduced apoptosis, whereas its knockdown significantly increased apoptotic cell populations. **(E)** Cell cycle analysis indicated that Snhg5 knockdown induced G1 phase arrest and reduced the S-phase fraction, whereas overexpression promoted G1/S transition. **(F)** Wound healing assays demonstrated enhanced migration in MC38-F0 cells upon Snhg5 overexpression, and impaired migratory capacity in MC38-F3 cells following knockdown. **(G)** Transwell migration and invasion assays confirmed that Snhg5 facilitated motility and invasiveness in CRC cells. Snhg5 silencing significantly reduced both migratory and invasive capacities. Scale bars = 100 μm. Data are presented as mean ± SEM from at least three independent experiments. Statistical significance was determined using Student's *t*-test or ANOVA as appropriate. ∗*P* < 0.05, ∗∗*P* < 0.01, ∗∗∗*P* < 0.001.

To assess the functional impact of Snhg5 on colorectal cancer cell behavior, we performed gain- and loss-of-function assays in both F0 and F3 MC38 sublines. CCK-8 assays revealed that Snhg5 overexpression significantly enhanced proliferation in F0 cells, whereas its knockdown markedly reduced proliferation in F3 cells over a 96-h period (p < 0.01; Fig. 4A). Consistent with these findings, colony formation assays demonstrated increased clonogenic capacity upon Snhg5 overexpression, and decreased colony formation following Snhg5 silencing (p < 0.01; Fig. 4B). EdU incorporation assays further supported these observations, showing a higher percentage of EdU-positive cells in the Snhg5-overexpressing group and a significant reduction in EdU labeling in Snhg5-depleted F3 cells (p < 0.01; Fig. 4C). Annexin V–FITC/PI staining revealed that Snhg5 knockdown significantly increased apoptotic cell populations in F3 cells (p < 0.001), while overexpression in F0 cells led to reduced apoptosis (p < 0.01; Fig. 4D). In addition, flow cytometric cell cycle analysis indicated that Snhg5 silencing induced G1 phase arrest and reduced S-phase fraction, whereas Snhg5 overexpression promoted G1/S transition (p < 0.01; Fig. 4E). To evaluate the effect of Snhg5 on cell motility, wound healing assays showed that knockdown of Snhg5 significantly impaired migratory ability in F3 cells, while its overexpression enhanced wound closure in F0 cells (p < 0.05; Fig. 4F). Transwell assays confirmed that both migration and invasion were suppressed by Snhg5 silencing and promoted by its overexpression (p < 0.01; Fig. 4G). Together, these results indicate that Snhg5 facilitates colorectal cancer cell proliferation, cell cycle progression, and motility while concurrently suppressing apoptosis, supporting its functional role in driving aggressive tumor phenotypes.

### 3.5 Snhg5 directly interacts with GNB2 in colorectal cancer cells

**Fig. 5:**
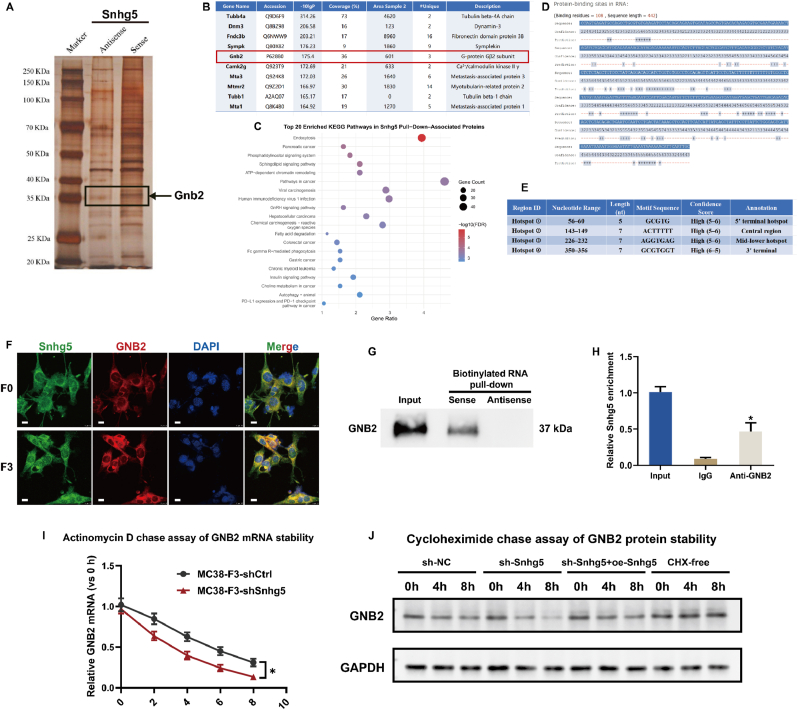
**GNB2 is identified as a direct RNA-binding partner of Snhg5 in CRC cells**. **(A)** Silver staining of biotinylated RNA pull-down eluates revealed a distinct enrichment pattern between sense and antisense groups. All enriched protein lanes were uniformly excised after entering the resolving gel (∼1 cm) and subjected to in-gel digestion for LC–MS/MS analysis. **(B)** Mass spectrometry identified GNB2 (G protein β2 subunit) as one of the top-ranked Snhg5-binding proteins based on peptide coverage (36 %), spectral abundance, and confidence score (−10lgP = 175.4). **(C)** KEGG pathway enrichment of Snhg5-interacting proteins indicated strong clustering in cancer-related signaling pathways, including colorectal cancer, phosphatidylinositol signaling, and choline metabolism. **(D**–**E)** In silico binding prediction using PRIdictor identified four high-confidence GNB2-binding motifs within the full-length Snhg5 transcript, located at nucleotide positions 56–60, 143–149, 226–232, and 350–356, with confidence scores of 5–6. **(F)** Dual-color RNA–FISH combined with immunofluorescence showed cytoplasmic colocalization of Snhg5 (green) and GNB2 (red) in both MC38-F0 and MC38-F3 cells, with stronger overlap observed in F3 cells. Nuclei were stained with DAPI (blue). Scale bars = 10 μm. **(G)** Western blot of pull-down products confirmed that endogenous GNB2 specifically bound to the sense strand of Snhg5 but not to the antisense control. **(H)** RNA immunoprecipitation using anti-GNB2 antibody demonstrated significant enrichment of Snhg5 compared with IgG control, validating the direct interaction. **(I)** Actinomycin D chase assay showing that Snhg5 knockdown accelerates the decay of Gnb2 mRNA, resulting in a reduced transcript half-life compared with shCtrl cells. **(J)** Cycloheximide chase assay demonstrating that depletion of Snhg5 decreases the stability of GNB2 protein, whereas Snhg5 re-expression partially restores GNB2 protein levels over time; GAPDH served as a loading control. Data are presented as mean ± SEM from three independent experiments. ∗*P* < 0.05 (Student's *t*-test or two-way ANOVA as appropriate).

To identify protein interactors of Snhg5, biotinylated RNA pull-down assays were performed using full-length sense and antisense transcripts of the murine Snhg5-203 isoform. Silver staining revealed a distinct ∼37 kDa band specifically enriched in the sense group, which was excised and analyzed by LC–MS/MS (Fig. 5A). Among the identified candidates, GNB2 (G-protein β2 subunit) ranked highly based on peptide coverage (36 %), spectral abundance, and identification confidence (−10lgP = 175.4), indicating a specific association (Fig. 5B). KEGG enrichment of Snhg5-associated proteins revealed significant clustering in cancer-related categories, including colorectal cancer, pancreatic cancer, and choline metabolism in cancer. Additional enrichment was observed in phosphatidylinositol, sphingolipid, and GnRH signaling pathways, as well as endocytosis, chromatin remodeling, and chemical carcinogenesis involving reactive oxygen species (Fig. 5C), suggesting functional relevance of the Snhg5 interactome in oncogenic signaling. To further investigate RNA–protein recognition, in silico binding prediction using PRIdictor identified 108 putative binding residues across the 442-nt Snhg5 transcript. Four discrete high-confidence motifs were localized at nucleotides 56–60 (GCGTG), 143–149 (ACTTTTT), 226–232 (AGGTGAG), and 350–356 (GCGTGGT), each scoring within the 5–6 range (Fig. 5D and E), consistent with known RNA–protein interaction motifs. Dual-color RNA-FISH combined with immunofluorescence confirmed cytoplasmic colocalization of Snhg5 and GNB2 in both F0 and F3 MC38 cells, with increased overlap observed in the metastatic F3 subline (Fig. 5F). Biotin-RNA pull-down followed by immunoblotting validated specific retrieval of endogenous GNB2 by the sense, but not antisense, transcript (Fig. 5G). RIP assays using anti-GNB2 antibody further demonstrated significant enrichment of Snhg5 compared to IgG control (p < 0.05), confirming a direct physical interaction (Fig. 5H). To clarify the functional consequence of this binding, we next assessed Gnb2 transcript and protein turnover. Actinomycin D chase assays revealed that Snhg5 knockdown significantly accelerated the decay of Gnb2 mRNA, leading to a shorter half-life compared with control cells (Fig. 5I). Consistently, cycloheximide chase experiments showed that depletion of Snhg5 reduced the stability of GNB2 protein, whereas re-expression of Snhg5 partially restored GNB2 protein levels over time (Fig. 5J). Together, these findings establish GNB2 as a direct RNA-binding partner of Snhg5 in colorectal cancer cells and indicate that Snhg5 enhances GNB2 expression at least in part by stabilizing both Gnb2 mRNA and GNB2 protein, supporting the presence of a functional Snhg5–GNB2 RNA–protein axis potentially involved in metastatic signaling regulation.

### 3.6 GNB2 is upregulated in colorectal cancer and correlates with poor prognosis

**Fig. 6:**
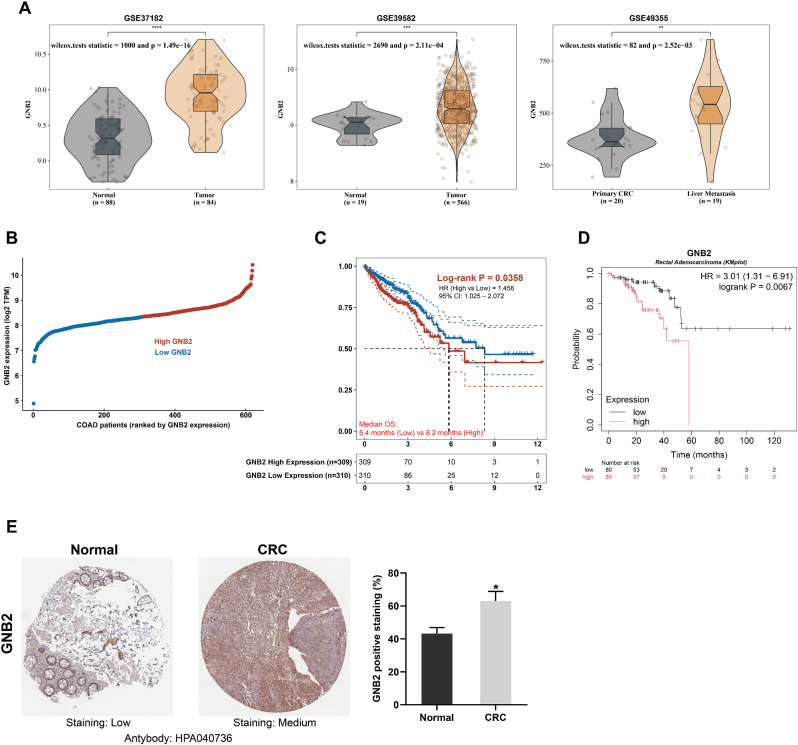
**GNB2 is upregulated in colorectal cancer and predicts poor prognosis**. **(A)** Violin plots from three independent GEO datasets consistently demonstrated elevated GNB2 mRNA levels in CRC tissues compared to adjacent normal mucosa (GSE37182 and GSE35982), with further upregulation observed in liver metastases relative to primary CRC tumors (GSE49355). **(B)** Expression distribution across TCGA-COAD patients showed substantial interpatient heterogeneity, with a subset exhibiting markedly high GNB2 expression. **(C)** Kaplan–Meier survival analysis based on TCGA-COAD data revealed that high GNB2 expression was significantly associated with shorter overall survival (median OS: 5.4 vs. 8.2 months; HR = 1.468; 95 % CI: 1.025–2.072; P = 0.0358). **(D)** Independent validation using the Kaplan–Meier Plotter for rectal adenocarcinoma confirmed the prognostic significance of GNB2, with high expression correlating with worse survival (HR = 3.01; 95 % CI: 1.31–6.91; P = 0.0067). **(E)** Representative IHC images from the Human Protein Atlas (HPA; antibody HPA040736) showed low cytoplasmic GNB2 staining in normal colonic epithelium and moderate staining in CRC tissues. Quantitative analysis revealed a significantly higher percentage of GNB2-positive cells in tumor tissues compared to normal controls. Data are presented as mean ± SEM. ∗*P* < 0.05 (Student's *t*-test).

To evaluate the clinical relevance of GNB2 in CRC, we analyzed its expression across multiple publicly available transcriptomic datasets. Violin plot analyses from three independent GEO cohorts (GSE37182, GSE35982, and GSE49355) consistently demonstrated significantly elevated GNB2 mRNA levels in CRC tissues relative to adjacent normal mucosa. Notably, the highest expression was observed in liver metastases compared to primary tumors (all p < 0.01; Fig. 6A). In the TCGA-COAD cohort, GNB2 expression levels varied substantially among patients, with a distinct subgroup showing pronounced overexpression (Fig. 6B). We next assessed the prognostic impact of GNB2 using two independent cohorts. In TCGA-COAD, high GNB2 expression was significantly associated with reduced overall survival (median OS: 5.4 vs. 8.2 months; HR = 1.468; p = 0.0358; Fig. 6C). Moreover, survival analysis performed through the Kaplan–Meier Plotter platform (https://kmplot.com) in a rectal adenocarcinoma-specific dataset further confirmed that elevated GNB2 expression predicted worse outcomes, with a hazard ratio of 3.01 (95 % CI, 1.31–6.91; log-rank p = 0.0067; Fig. 6D). To confirm GNB2 expression at the protein level, we examined immunohistochemical staining data from the Human Protein Atlas (HPA; antibody HPA040736). IHC analysis revealed low GNB2 expression in normal colonic epithelium and moderate cytoplasmic staining in CRC tissues. Quantification showed a significantly higher proportion of GNB2-positive cells in tumor tissues compared with normal controls (p < 0.05; Fig. 6E). Collectively, these results identify GNB2 as a consistently upregulated gene in CRC and a negative prognostic indicator. Its overexpression in metastatic tissues and strong association with adverse outcomes highlight GNB2 as a potential biomarker and therapeutic candidate in advanced CRC.

### 3.7 GNB2 overexpression rescues the anti-tumor effects of Snhg5 knockdown in highly metastatic CRC cells

**Fig. 7:**
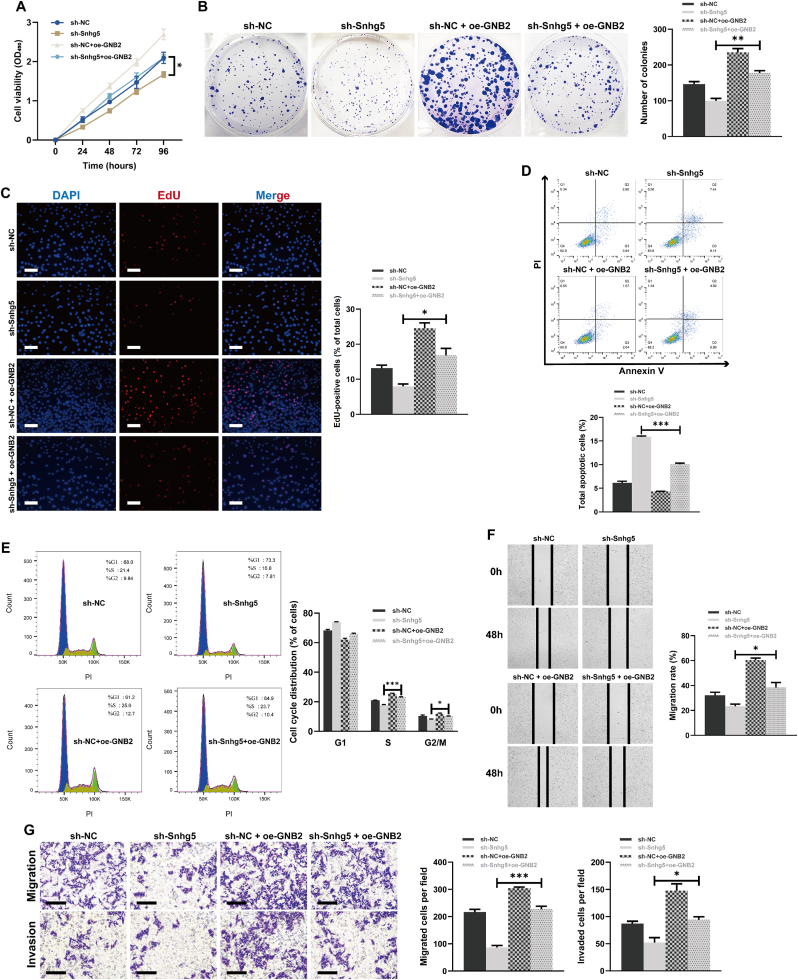
**GNB2 overexpression functionally rescues the tumor-suppressive effects of Snhg5 knockdown in highly metastatic CRC cells**. **(A)** CCK-8 assay showed that Snhg5 knockdown significantly suppressed cell viability over a 96-h period in MC38-F3 cells, whereas GNB2 overexpression partially restored proliferation. **(B)** Colony formation assays demonstrated that the reduction in clonogenicity induced by Snhg5 knockdown was significantly reversed by GNB2 overexpression. **(C)** EdU incorporation assay revealed that DNA synthesis was markedly decreased following Snhg5 silencing and partially rescued upon GNB2 overexpression. Scale bars = 100 μm. **(D)** Annexin V–FITC/PI apoptosis assay showed that Snhg5 depletion significantly increased apoptotic cell populations, which were reduced by GNB2 overexpression. **(E)** Cell cycle analysis revealed that Snhg5 knockdown induced G1 phase arrest and decreased the S-phase fraction, whereas co-expression of GNB2 alleviated these changes. **(F)** Wound healing assays demonstrated that the impaired migration capacity caused by Snhg5 silencing was significantly restored by GNB2 overexpression at 48 h. **(G)** Transwell migration and Matrigel-coated invasion assays further confirmed that GNB2 overexpression rescued the reduction in migratory and invasive abilities induced by Snhg5 knockdown. Scale bars = 100 μm. Data are presented as mean ± SEM from at least three independent experiments. ∗*P* < 0.05, ∗∗*P* < 0.01, ∗∗∗*P* < 0.001 (one-way ANOVA with Tukey's post hoc test unless otherwise indicated).

To elucidate whether GNB2 mediates the oncogenic function of Snhg5, we performed rescue assays in F3 cells following Snhg5 knockdown with or without GNB2 overexpression. CCK-8 assays demonstrated that silencing Snhg5 significantly suppressed cell viability over time, whereas reintroduction of GNB2 partially restored this proliferative capacity (Fig. 7A). Similarly, colony formation assays showed a marked reduction in the number of colonies after Snhg5 depletion, which was significantly reversed by GNB2 overexpression (Fig. 7B). EdU incorporation analysis confirmed decreased DNA synthesis upon Snhg5 knockdown, and this was partially rescued by GNB2 (Fig. 7C). Annexin V–FITC/PI staining revealed that the increase in apoptotic cell population induced by Snhg5 knockdown was attenuated by GNB2 restoration (Fig. 7D). In cell cycle analyses, Snhg5 depletion led to G1 phase arrest and a reduction in the S-phase fraction, whereas GNB2 overexpression alleviated these changes (Fig. 7E). Furthermore, wound healing and Transwell assays consistently showed that impaired migratory and invasive capabilities caused by Snhg5 knockdown were significantly reversed by GNB2 (Fig. 7F and G). Collectively, these findings demonstrate that GNB2 acts as a functional downstream effector of Snhg5, mediating its regulatory effects on proliferation, apoptosis resistance, and motility in highly metastatic CRC cells.

### 3.8 GNB2 overexpression reverses the inhibitory effects of Snhg5 knockdown on CRC liver metastasis in vivo

**Fig. 8:**
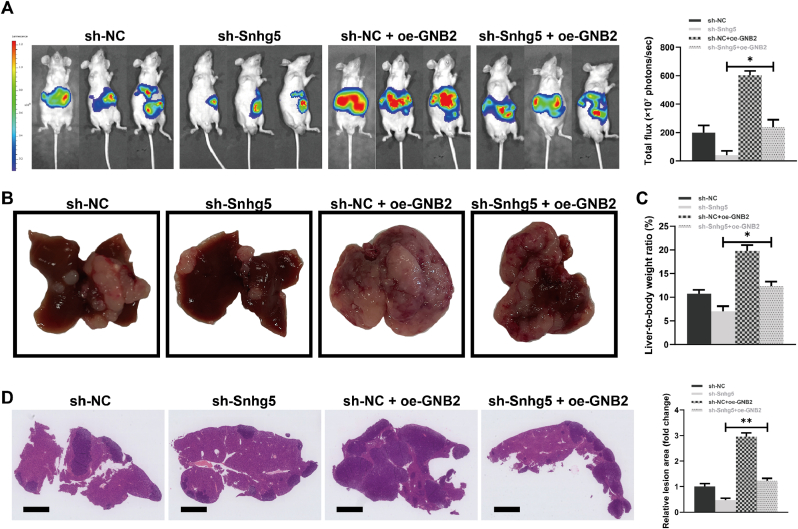
**GNB2 overexpression reverses the inhibitory effects of Snhg5 knockdown on CRC liver metastasis in vivo**. **(A)** Bioluminescence imaging performed on day 30 post-intrasplenic injection revealed that Snhg5 knockdown significantly reduced hepatic tumor burden in nude mice, whereas GNB2 overexpression markedly enhanced metastasis. Co-expression of GNB2 in Snhg5-depleted cells partially restored metastatic capacity. Quantification of total liver-associated photon flux (photons/sec) confirmed these trends. **(B)** Representative gross liver images showed fewer and smaller metastatic nodules in the sh-Snhg5 group, extensive tumor burden in the sh-NC + oe-GNB2 group, and intermediate phenotypes in the dual-modulated group. **(C)** Liver-to-body weight (LW/BW) ratios were significantly decreased in the sh-Snhg5 group and increased upon GNB2 overexpression. The dual intervention group partially restored the elevated LW/BW ratio. **(D)** H&E staining of liver tissues revealed widespread metastatic infiltration in GNB2-overexpressing mice, minimal tumor involvement following Snhg5 knockdown, and partial rescue of the metastatic phenotype in the co-expression group. Quantification of metastatic lesion area (relative fold change) corroborated the imaging and gross morphological findings. Scale bars = 1 mm. Data are presented as mean ± SEM (n = 5 per group). ∗*P* < 0.05, ∗∗*P* < 0.01 (one-way ANOVA with Tukey's post hoc test).

To determine whether GNB2 functions as a critical downstream mediator of Snhg5 in promoting CRC liver metastasis, we conducted an in vivo rescue experiment using a splenic injection model. MC38 cells with stable Snhg5 knockdown, GNB2 overexpression, or combined dual manipulation were inoculated into nude mice. Bioluminescence imaging at day 30 post-injection revealed that Snhg5 depletion markedly reduced hepatic tumor burden compared with controls, whereas GNB2 overexpression significantly enhanced liver metastasis. Importantly, GNB2 overexpression restored the metastatic capacity in Snhg5-deficient cells (Fig. 8A). Quantitative analysis of total photon flux showed a significant increase in the Snhg5-knockdown plus GNB2-overexpression group compared with Snhg5-knockdown alone (p < 0.05). Gross morphology of liver tissues further supported these findings. Mice in the Snhg5 knockdown group displayed fewer and smaller metastatic nodules, while those overexpressing GNB2 exhibited extensive and coalescent lesions. Co-administration of GNB2 in the context of Snhg5 silencing partially reversed this phenotype (Fig. 8B). Consistently, the liver-to-body weight ratio, a surrogate marker of tumor load, was significantly elevated in the GNB2-overexpressing group and reduced upon Snhg5 silencing. Dual modification partially restored the elevated ratio (p < 0.05; Fig. 8C). Histopathological analysis with H&E staining revealed that GNB2 overexpression exacerbated the extent of liver infiltration, while Snhg5 knockdown attenuated lesion formation. Quantitative analysis of metastatic lesion area demonstrated that the inhibitory effect of Snhg5 knockdown was significantly reversed upon GNB2 re-expression (p < 0.01; Fig. 8D). Collectively, these data indicate that GNB2 is a functional downstream effector of Snhg5 that promotes CRC liver metastasis in vivo.

### 3.9 Snhg5 activates Wnt/β-catenin–mediated EMT via GNB2 in colorectal cancer cells

**Fig. 9:**
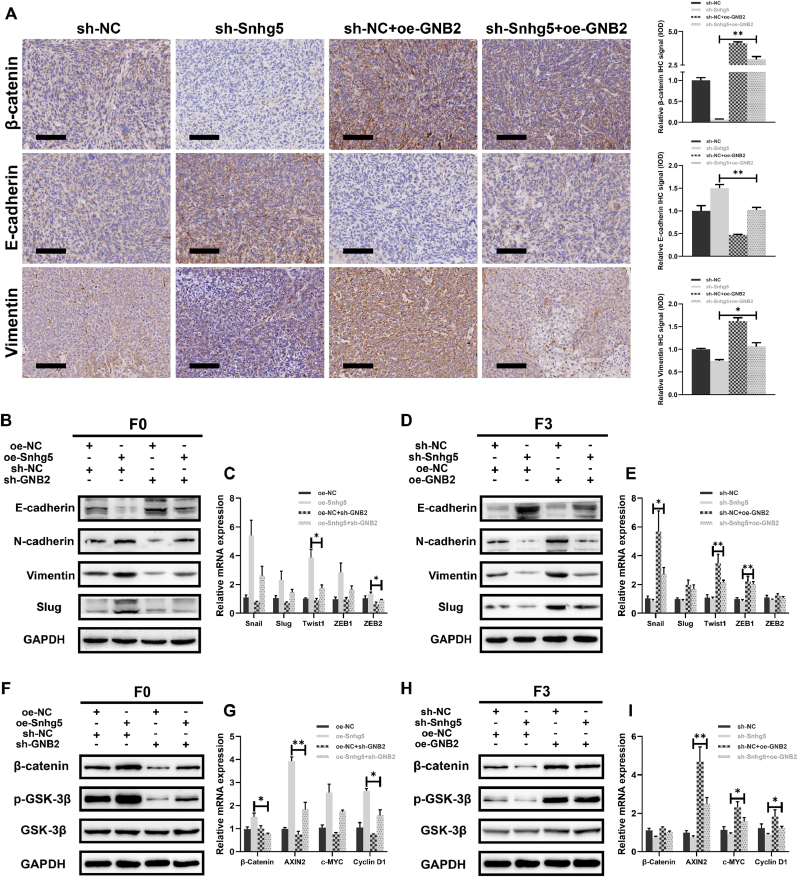
**The Snhg5–GNB2 axis promotes EMT and activates Wnt/β-catenin signaling in CRC cells**. **(A)** Immunohistochemistry of liver metastatic tissues revealed that Snhg5 knockdown reduced β-catenin and Vimentin expression while increasing E-cadherin levels. These changes were partially reversed by GNB2 overexpression. Quantification of IHC signal intensity (IOD) supported the observed protein alterations. Scale bars = 100 μm. **(B, D)** Western blot analysis of MC38-F0 (B) and MC38-F3 (D) cells showed that Snhg5 silencing increased E-cadherin expression while decreasing N-cadherin, Vimentin, and Slug levels. GNB2 overexpression reversed these EMT-associated changes. **(C, E)** RT-qPCR confirmed that knockdown of Snhg5 suppressed the transcription of key EMT regulators (Snail, Slug, Twist1, ZEB1, ZEB2), which was restored by GNB2 overexpression in both cell models. **(F, H)** Western blot analysis of Wnt signaling components revealed that Snhg5 knockdown reduced total β-catenin and phosphorylated GSK-3β (Ser9) levels, with no significant change in total GSK-3β. GNB2 overexpression reversed these effects in both MC38-F0 and F3 cells. **(G, I)** RT-qPCR analysis demonstrated that Snhg5 depletion led to reduced expression of canonical Wnt target genes (AXIN2, c-MYC, Cyclin D1), which was significantly restored by GNB2. Data are presented as mean ± SEM from at least three independent experiments. ∗*P* < 0.05, ∗∗*P* < 0.01, ∗∗∗*P* < 0.001 (one-way ANOVA with Tukey's post hoc test).

To elucidate the downstream mechanisms by which Snhg5 facilitates metastasis, we assessed whether the Snhg5/GNB2 axis regulates EMT and canonical Wnt/β-catenin signaling. Immunohistochemical analysis of liver metastatic tissues showed that Snhg5 knockdown markedly reduced β-catenin and Vimentin expression, while elevating E-cadherin levels, indicative of EMT suppression. These alterations were partially rescued by GNB2 overexpression (Fig. 9A). Consistent results were observed in vitro. Western blotting in both F0 and F3 cells demonstrated that Snhg5 silencing led to decreased expression of N-cadherin, Vimentin, and Slug, along with increased E-cadherin, all of which were reversed upon GNB2 restoration (Fig. 9B–D). At the transcriptional level, qRT-PCR confirmed that Snhg5 knockdown suppressed Snail, Slug, Twist1, ZEB1, and ZEB2, whereas GNB2 overexpression restored their expression (Fig. 9C–E). We further investigated whether this axis modulates Wnt/β-catenin pathway activation. In both cell models, Snhg5 knockdown reduced total β-catenin and phosphorylated GSK-3β levels, as well as downstream Wnt targets including AXIN2, c-MYC, and Cyclin D1. These effects were reversed by GNB2 overexpression (Fig. 9F–I), suggesting that GNB2 is required for Wnt/β-catenin activation downstream of Snhg5. Together, these findings indicate that the Snhg5/GNB2 axis promotes EMT and activates the Wnt/β-catenin pathway, thereby contributing to the metastatic phenotype of colorectal cancer cells.

## 4 Discussion

In this study, we systematically investigated the role of the lncRNA SNHG5 in promoting CRC liver metastasis and identified GNB2 as a novel downstream effector. Liver metastasis remains the most frequent and lethal form of distant spread in CRC, affecting nearly 50 % of patients and contributing to poor long-term survival, with 5-year survival rates falling below 15 % in unresectable cases [2]. Elucidating the molecular drivers underlying CRC liver metastasis is therefore essential for improving therapeutic strategies. Our findings offer three key insights. First, SNHG5 was consistently upregulated in CRC cells with high metastatic potential and in clinical liver metastases. Functional studies confirmed that SNHG5 promotes aggressive phenotypes, including enhanced proliferation, migration, and resistance to apoptosis. These results expand the understanding of SNHG5 beyond its previously reported roles in digestive system malignancies. Second, using RNA pull-down and mass spectrometry, we identified GNB2—a G protein β subunit—as a direct RNA-binding partner of SNHG5. Functional rescue assays demonstrated that GNB2 mediates SNHG5-induced tumor-promoting effects by activating the PI3K/AKT pathway, a canonical oncogenic axis. This is, to our knowledge, the first demonstration of a lncRNA directly modulating a G protein subunit to drive distant metastasis in solid tumors. Third, we propose that the SNHG5–GNB2 axis may contribute to metastatic colonization through coordinated regulation of Wnt/β-catenin signaling, immune evasion, and metabolic reprogramming. In our model, GNB2 links SNHG5 to AKT-mediated stabilization of β-catenin, facilitating EMT and activation of Wnt targets. This mechanistic framework provides a rationale for how lncRNA-mediated signaling convergence could establish a pro-metastatic immunometabolic niche. In summary, we have identified and characterized the SNHG5–GNB2 axis as a critical driver of CRC liver metastasis. By integrating lncRNA regulation, G protein signaling, and downstream oncogenic pathways, our findings expand the mechanistic landscape of metastatic CRC and offer new avenues for therapeutic targeting.

### 4.1 Aberrant upregulation of SNHG5 and its ceRNA-Mediated mechanism

SNHG5 (Small Nucleolar RNA Host Gene 5) is frequently overexpressed in various solid malignancies, including CRC, where its expression positively correlates with tumor size, metastatic burden, and pathological staging [[22], [23], [24],^39^]. In CRC tissues and cell lines, SNHG5 is consistently upregulated. Functional assays have demonstrated that SNHG5 promotes proliferation, migration, and apoptosis resistance in a stable manner [24,30,40]. In vitro and in vivo experiments have further confirmed that SNHG5 overexpression enhances the malignant phenotype of CRC cells, whereas its knockdown induces cell cycle arrest, increases apoptosis, and reduces migratory and invasive capacities. Mechanistically, SNHG5 functions primarily as a ceRNA, acting as a molecular sponge for tumor-suppressive miRNAs, thereby derepressing oncogenic transcripts and activating downstream signaling pathways. In CRC, SNHG5 competitively binds to miR-132-3p, alleviating its inhibition of CREB5, a transcription factor that facilitates CRC cell migration and invasion [30]. CREB5 further promotes EMT by inducing mesenchymal markers such as Vimentin and N-cadherin, driving epithelial cells toward a mesenchymal phenotype [26]. This regulatory axis appears to be conserved across cancer types. In cervical cancer, SNHG5 represses miR-132 and upregulates SOX4, thereby promoting tumor proliferation and invasion, underscoring miR-132 as a shared regulatory node of SNHG5 across tumor contexts [41]. Similar ceRNA mechanisms have been reported in other cancers. For instance, in breast cancer, SNHG5 regulates proliferation through the SNHG5–miR-154-5p–PCNA axis [31]; in HCC, it sponges miR-26a-5p to relieve repression of GSK3β, leading to Wnt/β-catenin pathway activation and EMT induction [32]. Furthermore, pro-metastatic phenotypes driven by SNHG5–miRNA interactions have been observed in melanoma and osteosarcoma, where SNHG5 promotes invasion via the miR-26a-5p/TRPC3 axis [42]. In breast cancer, SNHG5 activates a metabolic reprogramming program by regulating BACH1 through miR-299, thereby upregulating glycolytic enzymes such as HK2 and PFK1, and promoting proliferation via enhanced aerobic glycolysis [43]. Collectively, these findings indicate that SNHG5 promotes tumor progression through diverse ceRNA-dependent pathways that are both tissue-specific and functionally heterogeneous. The SNHG5–miR-132-3p–CREB5 axis in CRC exemplifies this regulatory mode and provides mechanistic insight into its role in EMT and metastasis.

### 4.2 SNHG5 interacts with RNA-binding proteins to regulate mRNA stability

Beyond its ceRNA activity, SNHG5 exerts critical regulatory functions by directly interacting with RNA-binding proteins (RBPs), thereby modulating mRNA stability and expression profiles. This mechanism has been implicated in multiple solid tumors, including CRC. In CRC cells, SNHG5 is predominantly localized in the cytoplasm, where it binds to the double-stranded RNA-binding protein Staufen1 (STAU1) and inhibits the Staufen-mediated mRNA decay (SMD) pathway. Damas et al. first reported that SNHG5 forms a complex with STAU1 and its target transcripts—such as SPATS2—disrupting recognition of mRNA structural elements and preventing degradation, thereby prolonging mRNA half-life [24]. Functional assays showed that knockdown of SNHG5 led to downregulation of multiple survival-associated mRNAs and increased apoptosis, underscoring its role in promoting cell survival through mRNA stabilization [24]. A similar mechanism was observed in HCC, where SNHG5 was found to interact with UPF1, a key component of RNA surveillance machinery. Li et al. demonstrated that SNHG5 binding to UPF1 impairs its degradation activity, resulting in accumulation of β-catenin and Cyclin D1, thereby activating the Wnt/β-catenin pathway, maintaining cancer stemness, and promoting self-renewal [27]. In acute myeloid leukemia (AML), SNHG5 interacts with PTBP1, stabilizing ATG5 mRNA and inducing autophagy in mesenchymal stromal cells (MSCs), which in turn acquire a cancer-associated fibroblast (CAF)-like phenotype, ultimately enhancing chemoresistance [44]. This expands the role of SNHG5 as a regulator of RBP-mediated mRNA stability beyond epithelial tumors. In papillary thyroid carcinoma, SNHG5 binds and stabilizes RBM47, preventing ubiquitin-mediated degradation of FOXO3 via recruitment of USP21. This leads to enhanced nuclear localization and transcriptional activity of FOXO3, thereby promoting autophagy through activation of ATG3/ATG5 expression [39]. Importantly, SNHG5–protein interactions appear to exhibit tissue-specific functionality and directional variability. In gastric cancer, SNHG5 has been reported to function as a tumor suppressor. Zhao et al. showed that SNHG5 binds to the epigenetic regulator MTA2 and sequesters it in the cytoplasm, thus preventing its nuclear translocation and participation in NuRD complex-mediated chromatin remodeling, resulting in inhibition of EMT and tumor migration [33]. This mechanism highlights a noncanonical mode of spatial sequestration that regulates protein function through subcellular localization. In summary, SNHG5 modulates post-transcriptional gene regulation by interacting with diverse RBPs such as STAU1, UPF1, and MTA2, thereby influencing mRNA decay, stabilization, and transcriptional activation. These mechanisms provide an additional regulatory layer beyond ceRNA function, underscoring the multifaceted roles of SNHG5 in driving metastatic behavior in a context-dependent manner.

### 4.3 Regulatory role of SNHG5 in EMT and Wnt/β-catenin signaling

EMT is a key process through which epithelial tumor cells acquire invasive and metastatic capabilities. It is characterized by the loss of apical–basal polarity, reduced expression of epithelial markers such as E-cadherin, and increased expression of mesenchymal markers including Vimentin and N-cadherin [45]. In CRC, EMT enables cancer cells to penetrate the basement membrane, enter the circulation, and initiate distant metastasis [12,46]. Several studies have demonstrated that SNHG5 promotes tumor progression by inducing EMT [30,32]. Consistent with previous findings, our study observed that high SNHG5 expression in CRC cells is associated with a classic EMT phenotype, including E-cadherin downregulation and upregulation of N-cadherin and Vimentin. Silencing SNHG5 reversed these changes and impaired migration and invasion, supporting its role in EMT regulation. The Wnt/β-catenin pathway is a central signaling cascade involved in EMT and maintenance of cancer stemness [15,47]. It drives EMT by upregulating transcription factors such as Snail and ZEB1, while repressing epithelial genes like E-cadherin, and also activates stemness-related genes including CD44 and LGR5 [48]. In hepatocellular carcinoma (HCC), Li et al. reported that SNHG5 upregulates GSK3β by sponging miR-26a-5p, thereby stabilizing β-catenin and activating Wnt/β-catenin signaling to induce EMT [32]. This pathway also enhances glycolytic metabolism (Warburg effect), supplying energy to support metastatic progression [49]. In our CRC model, we observed that SNHG5 upregulation significantly increased GNB2 expression. GNB2 is known to activate the PI3K/AKT–GSK3β–β-catenin axis, thereby indirectly enhancing Wnt signaling. These findings suggest that SNHG5 may regulate β-catenin stability through a noncanonical GNB2-dependent mechanism, linking SNHG5 to EMT induction via downstream β-catenin activation. Functional rescue assays further demonstrated that GNB2 knockdown attenuated SNHG5-induced EMT phenotypes, reinforcing the mechanistic connection. It is worth noting that the role of SNHG5 in EMT and Wnt signaling may be context-dependent. In contrast to its EMT-promoting role in CRC and HCC, SNHG5 has been reported to suppress EMT in gastric cancer by binding and retaining MTA2 in the cytoplasm, thereby inhibiting NuRD complex–mediated chromatin remodeling [33]. In non-small cell lung cancer, SNHG5 expression is downregulated, and its overexpression inhibits TGF-β1–induced EMT, suppressing cell migration and invasion [50]. Collectively, these data suggest that the functional impact of SNHG5 on EMT and Wnt/β-catenin signaling is tumor type–specific, likely determined by the cellular context, interaction networks, and microenvironmental cues. In CRC—a malignancy heavily dependent on EMT–stemness axis during metastasis—SNHG5 acts as a robust EMT inducer and Wnt activator. This dual role provides a mechanistic foundation for the functional integration of its downstream effector, GNB2, within oncogenic signaling modules.

### 4.4 GNB2 as a functional downstream effector of SNHG5 and a central node in signal integration

In this study, RNA pull-down combined with mass spectrometry identified GNB2 (G protein subunit beta 2) as a novel RNA-binding partner of SNHG5. Subsequent rescue experiments validated GNB2 as a key downstream effector mediating the pro-metastatic functions of SNHG5. GNB2, which encodes the β-subunit of heterotrimeric G proteins (Gαβγ), plays a central role in G protein–coupled receptor (GPCR) signaling and modulates multiple cellular processes, including proliferation, survival, motility, and metabolic reprogramming via pathways such as PI3K/AKT, MAPK, and sphingolipid signaling [35,51]. Although the oncogenic role of GNB2 has been reported in hematologic malignancies [52], its function in solid tumors, particularly CRC, remains poorly characterized. Here, we demonstrate through RIP, RNA pull-down, and immunofluorescence co-localization that SNHG5 directly binds GNB2 and that both are co-upregulated in highly metastatic CRC cells (F3). Functionally, SNHG5 knockdown significantly suppressed CRC cell proliferation and migration, while GNB2 overexpression partially rescued these phenotypes, supporting its role as a functional downstream mediator. Mechanistically, we show that the SNHG5–GNB2 axis activates the PI3K/AKT pathway and upregulates phosphorylated GSK3β, thereby stabilizing β-catenin and activating canonical Wnt/β-catenin signaling. In line with this, actinomycin D and cycloheximide chase assays indicate that SNHG5 directly prolongs the half-life of both Gnb2 mRNA and GNB2 protein, providing a concrete mechanistic link between RNA–protein binding and increased GNB2 abundance. These findings position GNB2 as a central molecular node linking SNHG5 to the coordinated activation of the AKT and Wnt pathways. This signal integration function of GNB2 is supported by prior studies in breast cancer, where it was shown to be a direct target of miR-142-3p; GNB2 knockdown activated AKT/mTOR signaling, altered autophagic flux, and modulated chemotherapeutic sensitivity [53]. Similar roles have been reported in hepatocellular carcinoma and other malignancies [27,31], although the SNHG5–GNB2–Wnt axis has not previously been systematically explored in CRC. Importantly, PI3K/AKT signaling is intricately linked to chemotherapy resistance, metabolic adaptation, and immune evasion [54], suggesting that the SNHG5–GNB2 axis may have broader implications beyond EMT regulation. In this context, GNB2 not only transduces oncogenic signals downstream of SNHG5, but also functions as a hub that coordinates multiple pro-metastatic pathways. In conclusion, GNB2 acts as a direct SNHG5-interacting protein and a critical downstream effector that mediates key malignant traits of CRC cells. The establishment of the SNHG5–GNB2 signaling axis expands the mechanistic repertoire of lncRNA-mediated regulation of classical oncogenic pathways and provides a compelling rationale for its consideration as a therapeutic target in metastatic CRC.

### 4.5 Potential role of the SNHG5–GNB2 axis in immune evasion, metabolic reprogramming, and Tumor Microenvironment Remodeling

Liver metastasis in CRC is not solely driven by intrinsic changes in tumor cells but also requires a permissive microenvironment at the distant metastatic site. Increasing evidence suggests that immune evasion, metabolic reprogramming, and the formation of a pre-metastatic niche (PMN) are essential components enabling successful colonization of disseminated tumor cells [16,55,56]. Our findings indicate that the SNHG5–GNB2 axis may regulate these processes through multiple interconnected mechanisms. (1) Immune Evasion: Evasion of immune surveillance is critical for tumor cell survival and metastatic seeding. The Wnt/β-catenin signaling pathway has been implicated in immune evasion across various solid tumors by inhibiting dendritic cell (DC) recruitment, reducing CD8^+^ T cell infiltration, and inducing PD-L1 expression [17,57]. Our data suggest that the SNHG5–GNB2 axis activates Wnt signaling, which may endow CRC cells with an immune “cold” phenotype. In parallel, the PI3K/AKT pathway—also activated by SNHG5–GNB2—can upregulate immunosuppressive cytokines such as IL-10 and TGF-β via the mTOR–STAT3 axis, further attenuating antitumor immunity. While immune cell profiling was not directly performed in this study, the upregulation of SNHG5 likely contributes to an immunosuppressive hepatic niche that favors metastatic seeding. (2) Metabolic Reprogramming: Metabolic adaptation is a hallmark of metastatic competence. PI3K/AKT signaling can enhance aerobic glycolysis (Warburg effect) by upregulating glucose transporters (e.g., GLUT1) and key glycolytic enzymes (e.g., HK2, LDHA) [18,58]. SNHG5 has also been shown to enhance glycolytic flux in breast cancer through the miR-299/BACH1 axis, promoting the expression of glycolytic enzymes such as HK2, PFK1, and GAPDH [43]. In glioma, SNHG5 sponges miR-205 to upregulate E2F3, thereby increasing glucose uptake and lactate production [59]. These findings suggest a conserved role for SNHG5 in metabolic reprogramming across cancers. The activation of AKT by SNHG5–GNB2 may further augment this metabolic shift. Moreover, multi-omics data from our study indicated potential enrichment of sphingolipid signaling, including upregulation of S1P synthetases and S1P receptor activity. Sphingosine-1-phosphate (S1P) is a known promigratory lipid mediator that also modulates angiogenesis and immune suppression [14,60]. (3) Tumor Microenvironment Remodeling: Metastatic niche formation often precedes the arrival of tumor cells at distant organs. Hepatic stellate cells (HSCs), upon activation, secrete extracellular matrix components and inflammatory cytokines that remodel the liver microenvironment. SNHG5 has been reported to be highly expressed in HSCs, where it promotes activation via the Hippo/YAP pathway [61]. Furthermore, in breast cancer–associated fibroblasts (CAFs), SNHG5 stabilizes ZNF281 mRNA through an m6A-dependent mechanism, upregulating CCL2/CCL5 expression and activating p38 MAPK signaling, which enhances vascular permeability and neovascularization to facilitate PMN formation [62]. These data indicate that SNHG5 may function beyond tumor-intrinsic regulation and actively reshape stromal cell phenotypes to favor metastasis. In summary, the SNHG5–GNB2 axis promotes CRC liver metastasis not only by enhancing EMT and cellular motility but also by coordinating immune suppression, metabolic adaptation, and stromal remodeling via activation of the Wnt/β-catenin and PI3K/AKT signaling pathways. This multifaceted role underscores its potential as a systemic driver of metastasis. Future studies employing spatial transcriptomics and immunoprofiling are warranted to further delineate this regulatory network and to explore the therapeutic implications of targeting the SNHG5–GNB2 axis in metastatic CRC.

### 4.6 Comparison with previous studies and mechanistic advancements

This study systematically investigates the role of the SNHG5–GNB2 axis in CRC metastasis, providing several conceptual and mechanistic extensions beyond existing literature. The novelty of our findings can be summarized in three key aspects: (1) Context-dependent Oncogenic Function of SNHG5: Previous studies have revealed a degree of functional heterogeneity for SNHG5 across different malignancies. In digestive system cancers—including CRC, HCC, and pancreatic cancer—SNHG5 is generally overexpressed and promotes tumor progression [24,27,63]. However, in other contexts such as gastric and lung adenocarcinoma, SNHG5 expression is often reduced, and its overexpression may exhibit tumor-suppressive properties [33,50]. These discrepancies suggest that SNHG5 is a context-dependent lncRNA, whose biological function is shaped by tumor type–specific cellular environments and signaling landscapes. Our study consolidates its pro-metastatic role in CRC, highlighting the need to interpret lncRNA function within appropriate disease-specific frameworks rather than extrapolating across cancer types. (2) Identification of GNB2 as a Novel Protein Effector of SNHG5: To date, most mechanistic studies of SNHG5 have focused on ceRNA networks, such as miR-132-3p/CREB5, miR-26a-5p/GSK3β, miR-132/SOX4, and miR-154-5p/PCNA, or its interaction with RBPs like STAU1 and MTA2 [24,[30], [31], [32], [33],^41^]. Our work is the first to report GNB2 as a direct RNA-binding partner of SNHG5. Functional rescue experiments demonstrate that GNB2 mediates the downstream effects of SNHG5 on cell migration, proliferation, and apoptosis resistance. This finding expands the functional repertoire of SNHG5, linking it to G protein signaling. More importantly, it reveals a multi-layered regulatory architecture, where SNHG5 engages both ceRNA-based and protein-based pathways to coordinate pro-metastatic outputs. (3) Expansion of SNHG5-Associated Phenotypes to Immune and Metabolic Regulation: Most previous studies have primarily focused on the role of SNHG5 in modulating classical tumor phenotypes such as proliferation, apoptosis, and invasion. In contrast, our study provides evidence that SNHG5 also influences immune evasion, metabolic reprogramming, and TME remodeling. Through activation of the Wnt/β-catenin and PI3K/AKT pathways, the SNHG5–GNB2 axis appears to orchestrate a shift toward an immunosuppressive “cold” TME phenotype in CRC. This integrative role positions SNHG5 as a central regulator within a “metastasis–immunity–metabolism” triad, offering a novel theoretical basis for considering SNHG5 as a co-target in immunotherapeutic strategies. In conclusion, this study enhances our understanding of SNHG5 by delineating a previously unrecognized signaling axis with GNB2, expanding its functional impact beyond traditional oncogenic pathways. By addressing both upstream regulatory complexity and downstream phenotypic diversity, we provide a comprehensive update to the current model of SNHG5 function in CRC metastasis and propose SNHG5–GNB2 as a multifaceted therapeutic target worthy of further translational investigation.

### 4.7 Clinical implications and future perspectives

Based on the integrated in vitro, in vivo and multi-cohort bioinformatic evidence generated here, the SNHG5–GNB2 axis emerges as a robust metastasis-associated module with clear translational relevance in CRC, particularly in the context of liver metastasis. A major strength of this work is the combination of mechanistic RNA–protein biochemistry, functional rescue experiments and clinically annotated datasets, which together support both the biological plausibility and the potential clinical generalizability of SNHG5–GNB2–driven metastatic behavior. At the biomarker level, SNHG5 is consistently upregulated in highly metastatic MC38 cells and in human liver metastases in our datasets, and prior studies have shown that SNHG5 is stably detectable in peripheral blood and can be packaged into exosomes [44]. In parallel, GNB2 overexpression is associated with adverse survival in independent CRC cohorts [64], and its protein expression can be readily quantified by immunohistochemistry. These convergent data suggest that quantitative assessment of SNHG5 and GNB2, alone or as a composite score, could help refine prognostic stratification and identify patients at high risk of liver metastasis who may benefit from intensified surveillance or earlier systemic intervention. In addition, the cytoplasmic localization and tumor-restricted overexpression of SNHG5 make it an attractive candidate for lncRNA-directed therapeutics, building on preclinical experience with antisense oligonucleotides against other oncogenic lncRNAs such as MALAT1 [65,66]. Combined targeting of SNHG5 and downstream GNB2–PI3K/AKT signaling—for example, by pairing RNA-based strategies with clinically available PI3K/AKT inhibitors [67]—may provide vertical blockade of this axis and delay metastatic outgrowth. Given the close linkage between SNHG5–GNB2 activity, Wnt/β-catenin signaling and immune exclusion [16,68], pharmacologic inhibition of this axis may convert “immune-cold” liver metastases into more inflamed, checkpoint-responsive lesions and thereby enhance the efficacy of anti–PD-1-based regimens. From a longer-term and more sustainable perspective, repurposing various natural compounds such as hinokitiol as prophylactic agents with immuno-modulatory effects and a favorable impact on cancer control [69] may complement SNHG5–GNB2-directed strategies, particularly in settings with limited access to complex biologics or in patients at high risk of recurrence after resection. Together, these considerations provide a rationale for incorporating SNHG5–GNB2 status into future biomarker-driven trials that test combinations of lncRNA-targeted therapeutics, pathway inhibitors and immunotherapy in metastatic CRC.

## 5 Limitations

This study has several limitations. First, although we now provide actinomycin D and cycloheximide chase assays indicating that SNHG5 stabilizes GNB2 mRNA and protein, respectively, we cannot exclude additional contributions from altered translation efficiency or ceRNA-mediated regulation. Second, all functional validation was performed in murine MC38 cells and immunodeficient or syngeneic mouse models, so extrapolation to human CRC requires confirmation in patient-derived organoids and xenografts. Third, the inferred links between SNHG5–GNB2 signaling, immune evasion and metabolic reprogramming were based on transcriptomic analyses rather than direct immune or metabolic profiling. Future studies should therefore define upstream transcriptional regulators of SNHG5, develop efficient liver-targeted delivery systems for SNHG5-directed oligonucleotides, and formally test rational combinations of SNHG5–GNB2 inhibition with PI3K/AKT blockade or immune checkpoint inhibitors in high-fidelity preclinical models.

## 6 Conclusion

This study provides the first comprehensive characterization of the oncogenic role of lncRNA SNHG5 in CRC liver metastasis. We demonstrated that SNHG5 promotes metastatic progression by directly binding to GNB2, a newly identified RNA-interacting protein, thereby stabilizing β-catenin and activating the canonical Wnt/β-catenin signaling pathway. This interaction induces EMT and facilitates distant dissemination. Functional assays in vitro and in vivo confirmed that the SNHG5–GNB2 axis enhances cell proliferation and migration while inhibiting apoptosis, contributing to an aggressive mesenchymal phenotype. Histopathological analyses and transcriptomic profiling from public datasets revealed that both SNHG5 and GNB2 are significantly upregulated in CRC primary tumors and liver metastases and are strongly associated with poor prognosis. Multi-omics data further suggest that this axis may coordinate immune evasion, metabolic adaptation, and remodeling of the metastatic niche. These findings expand the mechanistic repertoire of SNHG5 beyond classical ceRNA or RBP-dependent pathways, establishing it as a central node in signal integration during metastatic progression. Collectively, the SNHG5–GNB2 axis functions as a key regulatory module bridging oncogenic signaling, phenotypic plasticity, and tumor–microenvironment interaction. Its pivotal role in CRC metastasis provides a novel conceptual framework and lays a theoretical foundation for the development of targeted therapeutic strategies.

## CRediT authorship contribution statement

**Xinyi Chen:** Writing – original draft, Visualization, Validation, Methodology, Investigation, Formal analysis, Conceptualization. **Mu Yang:** Writing – review & editing, Visualization, Software, Formal analysis, Data curation. **Xiaoxiao Luo:** Writing – review & editing, Supervision, Resources, Project administration, Methodology. **Xianglin Yuan:** Writing – review & editing, Supervision, Project administration, Funding acquisition, Conceptualization.

## Ethical approval and consent to participate

All animal experiments in this study were approved by the Institutional Animal Care and Use Committee (IACUC) of Tongji Hospital, Tongji Medical College, Huazhong University of Science and Technology (Approval No. T1-202405039). All procedures were performed in strict accordance with institutional guidelines and the National Institutes of Health Guide for the Care and Use of Laboratory Animals. No human participants, identifiable human samples, or personally sensitive information were involved in this study. Publicly available datasets from TCGA and GEO were used in compliance with their corresponding data access and usage policies. Therefore, separate ethical approval or informed consent from patients was not required.

## Availability of data and materials

All data supporting the findings of this study are available in the article and its Supplementary Information. Public datasets analyzed include GEO (GSE14297, GSE37182, GSE44861, GSE71187, and GSE49355) and TCGA-COAD via UCSC Xena. Further information is available from the corresponding author upon reasonable request.

## Funding

This work was supported by the 10.13039/501100001809National Natural Science Foundation of China (Grant No. 82373522) and the China Postdoctoral Science Foundation (Grant No. 2024M761057).

## Declaration of competing interest

The authors declare that they have no known competing financial interests or personal relationships that could have appeared to influence the work reported in this paper.

## References

[1] Siegel R.L., Wagle N.S., Cercek A., Smith R.A., Jemal A. (2023). Colorectal cancer statistics, 2023. CA Cancer J. Clin..

[2] Zhou H., Liu Z., Wang Y. (2022). Colorectal liver metastasis: molecular mechanism and interventional therapy. Signal Transduct. Targeted Ther..

[3] Abd El Fattah Y.K., Abulsoud A.I., AbdelHamid S.G., AbdelHalim S., Hamdy N.M. (2023). CCDC144NL-AS1/hsa-miR-143-3p/HMGA2 interaction: In-silico and clinically implicated in CRC progression, correlated to tumor stage and size in case-controlled study; step toward ncRNA precision. Int. J. Biol. Macromol..

[4] Hamdy N.M., Zaki M.B., Rizk N.I. (2024). Unraveling the ncRNA landscape that governs colorectal cancer: a roadmap to personalized therapeutics. Life Sci..

[5] Rizk N.I., Kassem D.H., Abulsoud A.I. (2024). Revealing the role of serum exosomal novel long non-coding RNA NAMPT-AS as a promising diagnostic/prognostic biomarker in colorectal cancer patients. Life Sci..

[6] Takahashi H., Berber E. (2020). Role of thermal ablation in the management of colorectal liver metastasis. Hepatobiliary Surg. Nutr..

[7] Hamdy N.M., Basalious E.B., El-Sisi M.G. (2024). Advancements in current one-size-fits-all therapies compared to future treatment innovations for better improved chemotherapeutic outcomes: a step-toward personalized medicine. Curr. Med. Res. Opin..

[8] Nieto M.A., Huang R.Y., Jackson R.A., Thiery J.P. (2016). Emt: 2016. Cell.

[9] Anwar M.M., Albanese C., Hamdy N.M., Sultan A.S. (2022). Rise of the natural red pigment 'prodigiosin' as an immunomodulator in cancer. Cancer Cell Int..

[10] Hammad R., Aglan R.B., Mohammed S.A. (2022). Cytotoxic T cell expression of leukocyte-associated immunoglobulin-like Receptor-1 (LAIR-1) in viral hepatitis C-Mediated hepatocellular carcinoma. Int. J. Mol. Sci..

[11] Chen H., Zhai C., Xu X., Wang H., Han W., Shen J. (2023). Multilevel heterogeneity of colorectal cancer liver metastasis. Cancers (Basel).

[12] Liu X., Wang X., Yang Q. (2024). Th17 cells secrete TWEAK to trigger epithelial-mesenchymal transition and promote colorectal cancer liver metastasis. Cancer Res..

[13] Ding Y., Hao K., Li Z. (2020). c-Fos separation from Lamin A/C by GDF15 promotes colon cancer invasion and metastasis in inflammatory microenvironment. J. Cell. Physiol..

[14] Deng J., Liu Y., Lee H. (2012). S1PR1-STAT3 signaling is crucial for myeloid cell colonization at future metastatic sites. Cancer Cell.

[15] Zhao H., Ming T., Tang S. (2022). Wnt signaling in colorectal cancer: pathogenic role and therapeutic target. Mol. Cancer.

[16] Jiang W., Guan B., Sun H. (2025). WNT11 promotes immune evasion and resistance to Anti-PD-1 therapy in liver metastasis. Nat. Commun..

[17] Huang T.X., Tan X.Y., Huang H.S. (2022). Targeting cancer-associated fibroblast-secreted WNT2 restores dendritic cell-mediated antitumour immunity. Gut.

[18] Dong S., Liang S., Cheng Z. (2022). ROS/PI3K/Akt and Wnt/beta-catenin signalings activate HIF-1alpha-induced metabolic reprogramming to impart 5-fluorouracil resistance in colorectal cancer. J. Exp. Clin. Cancer Res..

[19] Lulli M., Napoli C., Landini I., Mini E., Lapucci A. (2022). Role of non-coding RNAs in colorectal cancer: focus on long non-coding RNAs. Int. J. Mol. Sci..

[20] Zhang C., Wang L., Jin C. (2021). Long non-coding RNA Lnc-LALC facilitates colorectal cancer liver metastasis via epigenetically silencing LZTS1. Cell Death Dis..

[21] Li Y.H., Hu Y.Q., Wang S.C., Li Y., Chen D.M. (2020). LncRNA SNHG5: a new budding star in human cancers. Gene.

[22] Huang Q., Xia Y.G., Huang Y.J. (2024). An increase in SNHG5 expression is associated with poor cancer prognosis, according to a meta-analysis. Eur. J. Med. Res..

[23] Pashirzad M., Sahebkar A. (2024). The prognostic value and clinical significance of lncRNA SNHG5 expression in patients with multiple malignancies: a bioinformatic and meta-analysis. Curr. Cancer Drug Targets.

[24] Damas N.D., Marcatti M., Come C. (2016). SNHG5 promotes colorectal cancer cell survival by counteracting STAU1-mediated mRNA destabilization. Nat. Commun..

[25] Liao Z., Nie H., Wang Y., Luo J., Zhou J., Ou C. (2021). The emerging landscape of long non-coding RNAs in colorectal cancer metastasis. Front. Oncol..

[26] Wang A., Yan S., Liu J. (2025). Endoplasmic reticulum stress-related super enhancer promotes epithelial-mesenchymal transformation in hepatocellular carcinoma through CREB5 mediated activation of TNC. Cell Death Dis..

[27] Li Y., Hu J., Guo D. (2022). LncRNA SNHG5 promotes the proliferation and cancer stem cell-like properties of HCC by regulating UPF1 and Wnt-signaling pathway. Cancer Gene Ther..

[28] Kang S., Ou C., Yan A. (2023). Long noncoding RNA SNHG5 induces the NF-kappaB pathway by regulating miR-181c-5p/CBX4 axis to promote the progression of non-small cell lung cancer. Arch. Bronconeumol..

[29] Wei S., Sun S., Zhou X. (2021). SNHG5 inhibits the progression of EMT through the ubiquitin-degradation of MTA2 in oesophageal cancer. Carcinogenesis.

[30] Zhang M., Li Y., Wang H., Yu W., Lin S., Guo J. (2019). LncRNA SNHG5 affects cell proliferation, metastasis and migration of colorectal cancer through regulating miR-132-3p/CREB5. Cancer Biol. Ther..

[31] Chi J.R., Yu Z.H., Liu B.W. (2019). SNHG5 promotes breast cancer proliferation by sponging the miR-154-5p/PCNA axis. Mol. Ther. Nucleic Acids.

[32] Li Y., Guo D., Zhao Y. (2018). Long non-coding RNA SNHG5 promotes human hepatocellular carcinoma progression by regulating miR-26a-5p/GSK3beta signal pathway. Cell Death Dis..

[33] Zhao L., Guo H., Zhou B. (2016). Long non-coding RNA SNHG5 suppresses gastric cancer progression by trapping MTA2 in the cytosol. Oncogene.

[34] Li M., Zhang Y.Y., Shang J., Xu Y.D. (2019). LncRNA SNHG5 promotes cisplatin resistance in gastric cancer via inhibiting cell apoptosis. Eur. Rev. Med. Pharmacol. Sci..

[35] Yoda A., Adelmant G., Tamburini J. (2015). Mutations in G protein beta subunits promote transformation and kinase inhibitor resistance. Nat. Med..

[36] Luo C., Xiao Z., Yang W. (2025). GNG2 inhibits brain metastases from colorectal cancer via PI3K/AKT/mTOR signaling pathway. Sci. Rep..

[37] Zhang L., Sahar A.M., Li C. (2022). A detailed multi-omics analysis of GNB2 gene in human cancers. Braz. J. Biol..

[38] Lu W., Pan X., Dai S. (2021). Identifying stage II colorectal cancer recurrence associated genes by microarray Meta-analysis and building predictive models with machine learning algorithms. J. Oncol..

[39] Qin Y., Sun W., Wang Z. (2022). RBM47/SNHG5/FOXO3 axis activates autophagy and inhibits cell proliferation in papillary thyroid carcinoma. Cell Death Dis..

[40] Wang B., Zhou Q., Cheng C.E. (2025). Long noncoding RNA SNHG5 promotes 5-fluorouracil resistance in colorectal cancer by regulating miR-26b/p-glycoprotein axis. World J. Gastrointest. Oncol..

[41] Zhang L., Wu X., Li Y., Teng X., Zou L., Yu B. (2021). LncRNA SNHG5 promotes cervical cancer progression by regulating the miR-132/SOX4 pathway. Autoimmunity.

[42] Gao J., Zeng K., Liu Y., Gao L., Liu L. (2019). LncRNA SNHG5 promotes growth and invasion in melanoma by regulating the miR-26a-5p/TRPC3 pathway. OncoTargets Ther..

[43] Huang S.L., Huang Z.C., Zhang C.J. (2022). LncRNA SNHG5 promotes the glycolysis and proliferation of breast cancer cell through regulating BACH1 via targeting miR-299. Breast Cancer.

[44] Song Y., Hu L., Cheng J., Li Z., Zheng J. (2025). LncRNA SNHG5 induces CAFs-like phenotype and autophagy of AML-MSCs via PTBP1/ATG5 axis to confer chemoresistance of AML cells. Cell. Signal..

[45] Dongre A., Weinberg R.A. (2019). New insights into the mechanisms of epithelial-mesenchymal transition and implications for cancer. Nat. Rev. Mol. Cell Biol..

[46] Zhang N., Ng A.S., Cai S., Li Q., Yang L., Kerr D. (2021). Novel therapeutic strategies: targeting epithelial-mesenchymal transition in colorectal cancer. Lancet Oncol..

[47] Tang Q., Chen J., Di Z. (2020). TM4SF1 promotes EMT and cancer stemness via the Wnt/beta-catenin/SOX2 pathway in colorectal cancer. J. Exp. Clin. Cancer Res..

[48] Xu X., Zhang M., Xu F., Jiang S. (2020). Wnt signaling in breast cancer: biological mechanisms, challenges and opportunities. Mol. Cancer.

[49] Zuo Q., He J., Zhang S. (2021). PPARgamma Coactivator-1alpha suppresses metastasis of hepatocellular carcinoma by inhibiting warburg effect by PPARgamma-Dependent WNT/beta-Catenin/Pyruvate dehydrogenase kinase isozyme 1 axis. Hepatology.

[50] Li Z., Wu Y., Zhang C. (2023). LncRNA SNHG5 suppresses cell migration and invasion of human lung adenocarcinoma via regulation of epithelial-mesenchymal transition. J. Oncol..

[51] Smrcka A.V. (2008). G protein betagamma subunits: central mediators of G protein-coupled receptor signaling. Cell. Mol. Life Sci..

[52] Kotani S., Yoda A., Kon A. (2019). Molecular pathogenesis of disease progression in MLL-rearranged AML. Leukemia.

[53] Shi Y., Wang J., Tao S. (2023). miR-142-3p improves paclitaxel sensitivity in resistant breast cancer by inhibiting autophagy through the GNB2-AKT-mTOR pathway. Cell. Signal..

[54] Stefani C., Miricescu D., Stanescu S. (2021). Growth factors, PI3K/AKT/mTOR and MAPK signaling pathways in colorectal cancer pathogenesis: where are we now?. Int. J. Mol. Sci..

[55] Li Y., Wang H., Mao D., Che X., Chen Y., Liu Y. (2025). Understanding pre-metastatic niche formation: implications for colorectal cancer liver metastasis. J. Transl. Med..

[56] Zhou J., Song Q., Li H. (2024). Targeting circ-0034880-enriched tumor extracellular vesicles to impede SPP1(high)CD206(+) pro-tumor macrophages mediated pre-metastatic niche formation in colorectal cancer liver metastasis. Mol. Cancer.

[57] Takeuchi Y., Tanegashima T., Sato E. (2021). Highly immunogenic cancer cells require activation of the WNT pathway for immunological escape. Sci. Immunol..

[58] Hoxhaj G., Manning B.D. (2020). The PI3K-AKT network at the interface of oncogenic signalling and cancer metabolism. Nat. Rev. Cancer.

[59] Li X., Liu L., Luo Y. (2019). Long non-coding RNA SNHG5 promotes glioma progression via miR-205/E2F3 axis. Biosci. Rep..

[60] Pyne N.J., Pyne S. (2010). Sphingosine 1-phosphate and cancer. Nat. Rev. Cancer.

[61] Zhang R., Zhan Y., Lang Z., Li Y., Zhang W., Zheng J. (2024). LncRNA-SNHG5 mediates activation of hepatic stellate cells by regulating NF2 and Hippo pathway. Commun. Biol..

[62] Zeng H., Hou Y., Zhou X. (2022). Cancer-associated fibroblasts facilitate premetastatic niche formation through lncRNA SNHG5-mediated angiogenesis and vascular permeability in breast cancer. Theranostics.

[63] Jamali E., Safarzadeh A., Hussen B.M., Liehr T., Ghafouri-Fard S., Taheri M. (2023). Single cell RNA-seq analysis with a systems biology approach to recognize important differentially expressed genes in pancreatic ductal adenocarcinoma compared to adjacent non-cancerous samples by targeting pancreatic endothelial cells. Pathol. Res. Pract..

[64] Tian J., Cao X., Jiang Z. (2024). LncRNA CCAT2 promotes the proliferation and metastasis of colorectal cancer through activation of the ERK and Wnt signaling pathways by regulating GNB2 expression. Cancer Med..

[65] Gutschner T., Hammerle M., Eissmann M. (2013). The noncoding RNA MALAT1 is a critical regulator of the metastasis phenotype of lung cancer cells. Cancer Res..

[66] Gong N., Teng X., Li J., Liang X.J. (2019). Antisense oligonucleotide-conjugated nanostructure-targeting lncRNA MALAT1 inhibits cancer metastasis. ACS Appl. Mater. Interfaces.

[67] Ma Y., Sender S., Sekora A. (2022). The inhibitory response to PI3K/AKT pathway inhibitors MK-2206 and Buparlisib is related to genetic differences in pancreatic ductal adenocarcinoma cell lines. Int. J. Mol. Sci..

[68] Luke J.J., Bao R., Sweis R.F., Spranger S., Gajewski T.F. (2019). WNT/beta-catenin pathway activation correlates with immune exclusion across human cancers. Clin. Cancer Res..

[69] Chiang Y.F., Huang K.C., Chen H.Y. (2024). Hinokitiol inhibits breast cancer cells in vitro stemness-progression and self-renewal with apoptosis and autophagy modulation via the CD44/Nanog/SOX2/Oct4 pathway. Int. J. Mol. Sci..

