# Supplementary material for: SNHG5 enhances colorectal cancer metastasis through RNA–protein interaction with GNB2 and activation of canonical Wnt signaling
Source: Noncoding RNA Res. 2026 Jan 2;17:128–49. doi: 10.1016/j.ncrna.2025.12.002 (PMC12809277; doi:10.1016/j.ncrna.2025.12.002)
Supplement: Multimedia component 1 [file mmc1.docx]

**Supplementary Table S1. Primer sequences for in vitro transcription of biotin-labeled RNA probes targeting mouse Snhg5.**

| **Probe type** | **Primer direction** | **Sequence (5′–3′)** | **Amplicon size** |
| --- | --- | --- | --- |
| **Sense strand** | Forward | TAATACGACTCACTATAGGGGCCTGCGTGTGAAGATCCTA | 64 bp |
|  | Reverse | TCCCCATTTTATTTCAATCACTGC |  |
| **Antisense strand** | Forward | GCCTGCGTGTGAAGATCCTA | 64 bp |
|  | Reverse | TAATACGACTCACTATAGGGTCCCCATTTTATTTCAATCACTGC |  |
