# Supplementary material for: SNHG5 enhances colorectal cancer metastasis through RNA–protein interaction with GNB2 and activation of canonical Wnt signaling
Source: Noncoding RNA Res. 2026 Jan 2;17:128–49. doi: 10.1016/j.ncrna.2025.12.002 (PMC12809277; doi:10.1016/j.ncrna.2025.12.002)
Supplement: Multimedia component 2 [file mmc2.docx]

**Supplementary Table S2. Validated shRNA sequences targeting murine Snhg5 and Gnb2.**

| **Gene** | **Ensembl Gene ID** | **shRNA ID** | **Target Sequence (sense, 5′→3′)** | **Target Region (RefSeq)** |
| --- | --- | --- | --- | --- |
| **Snhg5** | ENSMUSG00000097195 | shSnhg5‑1 | GCTCTTGAAGACCTCACCTA | NM_001164420.1, nt 481–501 |
|  |  | shSnhg5‑2 | GCCAGATGATCATTGACTTAA | nt 931–951 |
|  |  | shSnhg5‑3 | GCACTGTCCTTTGTTGAAGAT | nt 1273–1293 |
| **Gnb2** | ENSMUSG00000026070 | shGnb2‑1 | CCGCTCTTCGACAAGATGAAT | NM_010315.3, nt 201–221 |
|  |  | shGnb2‑2 | GCTGAATGTAGATAAGTACAA | nt 451–471 |
|  |  | shGnb2‑3 | GCTAGATGAAGAGAAGCTCAT | nt 640–660 |
